# Type 2 Inflammatory Diseases: The Crossroads of Immunity and Metabolism

**DOI:** 10.34133/research.1347

**Published:** 2026-07-08

**Authors:** Lin Chen, Zhou-Xian Pan, Zi-Qi Ma, Yu-Xuan Jin, Yi Ru, Pei-Song Gao, Cheng Yang, Shi-Su Zhao, Jing Zhang, Ji-Han Li, Christopher Chang, Yu-Zheng Zhao, Jin-Lyu Sun

**Affiliations:** ^1^ Department of Allergy, State Key Laboratory of Complex Severe and Rare Diseases, Peking Union Medical College Hospital, Chinese Academy of Medical Sciences and Peking Union Medical College, Beijing 100730, China.; ^2^ Optogenetics and Synthetic Biology Interdisciplinary Research Center, Shanghai Frontiers Science Center of Optogenetic Techniques for Cell Metabolism, State Key Laboratory of Bioreactor Engineering, School of Pharmacy, East China University of Science and Technology, Shanghai 200237, China.; ^3^ State Key Laboratory of Common Mechanism Research for Major Diseases, Department of Biochemistry and Molecular Biology, Medical Primate Research Center, Neuroscience Center, Institute of Basic Medical Sciences Chinese Academy of Medical Sciences, School of Basic Medicine Peking Union Medical College, Beijing 100005, China.; ^4^ Division of Allergy and Clinical Immunology, Johns Hopkins University School of Medicine, Baltimore, MD 21224, USA.; ^5^ Division of Immunology, Allergy and Rheumatology, Joe DiMaggio Children’s Hospital, Memorial Healthcare System, Hollywood, FL 33021, USA.

## Abstract

The understanding of type 2 inflammatory diseases is undergoing a paradigm shift from “immune imbalance” toward “immune–metabolic crosstalk”. Energy metabolism not only fuels immune responses but also fundamentally dictates the functional phenotypes of immune cells through metabolic reprogramming. By systematically integrating metabolic programming data of key immune cells across diverse tissues (skin, gut, nasal, and ocular mucosa), this review constructs a cross-disease “metabolism–signaling network” atlas. The synthesis highlights that glycolysis and mTORC1 signaling are predominantly coupled with the pro-inflammatory outputs of Th2 cells and ILC2s, whereas fatty acid oxidation (FAO) and oxidative phosphorylation (OXPHOS) sustain the homeostasis of Tregs and M2-like macrophages. Furthermore, this review characterizes how the tryptophan, glutamine, and arginine pathways fine-tune the immune tolerance boundary via the IDO–AhR–mTOR axis. We also elucidate a conserved inter-organ “hypoxia–HIF-1α–lactylation” axis, which, in conjunction with tissue-specific metabolic branches (e.g., the ceramide pathway in the skin, the SCFA circuit in the gut, and the lactate–GPR81 loop in the mucosa), collectively sculpts local microenvironments and remodeling trajectories. Ultimately, a novel diagnostic and therapeutic framework centered on metabolic phenotyping is proposed, providing prospective insights into targeting metabolic checkpoints for precision immunotherapy in type 2 inflammation.

## Introduction

Type 2 inflammatory diseases are characterized by high prevalence and marked heterogeneity, posing long-standing challenges to public health and clinical management. The research paradigm has progressed from identifying clinical phenotypes to elucidating immunologic mechanisms and, more recently, to systems-level integration and precision intervention. Early 20th-century insights into hay fever and anaphylaxis laid the groundwork for the allergic disease spectrum. Subsequently, the discovery of mast cells (MCs) and immunoglobulin E (IgE) established the immunological basis of immediate hypersensitivity. With the rise of molecular immunology, signaling pathways mediated by T helper type 2 (Th2) cells and IgE, together with type II inflammatory mechanisms, were systematically delineated, thereby driving the development and clinical deployment of targeted biologics such as anti-IgE, anti-interleukin-4 receptor α (IL-4Rα), and anti-IL-5 therapies [1,2].

Recent studies reveal that group 2 innate lymphoid cells (ILC2s), epithelial alarmins, thymic stromal lymphopoietin (TSLP), and IL-33, as well as tissue-resident memory cells, have shifted the research focus from downstream inflammation to upstream immune regulatory networks, catalyzing a new paradigm of immune–metabolic interactions [3]. Energy metabolism not only supplies substrates for immune responses but also serves as a pivotal signaling hub that determines cell fate and effector thresholds. The spectrum of type 2 inflammatory diseases is broad. Airway disorders include allergic rhinitis (AR), allergic asthma (AS), and chronic rhinosinusitis with nasal polyps (CRSwNP); skin disorders include atopic dermatitis (AD), chronic spontaneous urticaria (CSU), and delayed-type contact dermatitis (DCD); gastrointestinal disorders include food allergy (FA), eosinophilic esophagitis (EoE), and eosinophilic gastroenteritis (EGE); ocular disease includes allergic conjunctivitis (AC). In addition, drug allergy, anaphylaxis, aspirin-exacerbated respiratory disease (AERD), and occupational asthma (OA) represent systemic or special entities [4].

At the cellular scale, metabolic reprogramming spans sensitization, acute responses, and chronicity. Effector populations—including Th2 cells, ILC2s, eosinophils (Eos), MCs, and B cells/plasma cells (PCs)—commonly display up-regulated glycolysis and heightened oxidative stress to meet demands for rapid proliferation and high-throughput secretion [5]. By contrast, the metabolic states of dendritic cells (DCs), macrophages (Mφs), epithelial cells (ECs), and fibroblasts (FBs) dictate the direction and magnitude of antigen presentation, tissue repair, and structural remodeling in parallel. More specifically, glycolysis and mechanistic target of rapamycin complex 1 (mTORC1) signaling are frequently coupled to pro-inflammatory programs. Fatty acid oxidation (FAO) and oxidative phosphorylation (OXPHOS) support homeostasis in regulatory T cells (Tregs) and M2-like Mφs. Amino acid pathways—including tryptophan (Trp), glutamine (Gln), and arginine (Arg) metabolism—fine-tune the immune tolerance boundary via axes such as indoleamine 2,3-dioxygenase (IDO)–aryl hydrocarbon receptor (AhR)–mTOR [6]. Collectively, these “metabolic fingerprints” constitute the metabolic ecosystem of allergic inflammation and correlate closely with disease severity and therapeutic responsiveness.

Tissue-specific metabolic microenvironments further amplify such crosstalk. In the airways, a “hypoxia–hypoxia-inducible factor 1α (HIF-1α) stabilization–glycolytic enhancement–lactate accumulation” axis synergizes with epithelial alarmins, sculpting ILC2- and Th2-driven type II inflammation and steering smooth muscle and stromal remodeling [7]. In the skin, a coupled “lipidome–barrier–inflammation” triad—exemplified by imbalances in very-long-chain fatty acids and ceramide (CER) metabolism—disrupts the barrier and promotes transcutaneous sensitization. In the gut, short-chain fatty acids (SCFAs) and the indole–AhR–IL-22 axis bias oral antigen exposure toward tolerance versus allergy. In the nasal cavity and on the ocular surface, a cross-cellular glycolysis–lactate–G protein-coupled receptor 81 (GPR81) circuit associates with persistent inflammation and tissue remodeling. Convergent inter-organ evidence points to an actionable common axis “hypoxia–HIF-1α–glycolysis–lactate/lactylation signaling” that links multiple allergic phenotypes and interweaves with lipid and amino acid metabolic branches [8].

On this basis, a “metabolism–signaling” atlas of type 2 inflammatory diseases is taking shape. Single-cell and spatial omics have revealed both shared and disease-specific metabolic features: The former include widespread activation of core pathways such as the mTORC1–glycolysis axis; the latter include lipid peroxidation (LPO), Gln flux, phosphofructokinase-2/fructose-2,6-bisphosphatase 3 (PFKFB3)-dependent control of glycolysis, and GPR81-mediated lactate sensing—distinct metabolic hallmarks that inform “metabotype–phenotype” stratification and underpin the development of precise monitoring systems [9].

In parallel, translational advances indicate that metabolic intervention is moving from concept to clinical practice. Therapeutic strategies exemplified by leukotriene pathway inhibition provide a successful precedent; concurrently, antagonists of prostaglandin D₂ receptor 2 (CRTH2), activators of AMP-activated protein kinase (AMPK), inhibitors targeting mTORC1/HIF-1α, and cell-specific metabolic reprogramming approaches (e.g., nanoparticle- or liposome-based targeted delivery) are under active investigation. Targeted modulation of key metabolic nodes—such as PFKFB3, glutaminase 1 (GLS1), and GPR81—also shows encouraging druggability.

At the nutrition and lifestyle level, the mediterranean diet (MD), omega-3 (ω-3) long-chain polyunsaturated fatty acids (LC-PUFAs), high-fiber diets, and regular physical activity can promote SCFA production, remodel lipid metabolic profiles, and improve systemic metabolic homeostasis, thereby helping to reduce chronic inflammatory burden [10]. Meanwhile, microbiome-directed strategies—including probiotic/prebiotic supplementation, fecal microbiota transplantation (FMT), and the application of engineered “functional” microbes—are opening new avenues to rebuild the “microbiota–metabolism–immunity” axis. For companion diagnostics and biomarker development, metabolomic analyses of exhaled breath condensate (EBC) and volatile organic compounds (VOCs), together with plasma Trp–kynurenine ratio monitoring, and multimodal pipelines that integrate “electronic nose” technologies with machine learning, are accelerating clinical translation and precision assessment. To comprehensively resolve the multidimensional networks governing allergic inflammation—particularly by integrating recent discrete insights on spatial immunometabolic niches, epigenetic modifications such as histone lactylation, neural–immune–metabolic circuitry, and data-driven patient stratification—we here systematize an integrative framework of “metabolism–immunity–stroma–microbiota” [11]. Centering on metabolic programs in key immune cell populations—including T cells and ILC2s, Eos, MCs, B cells and PCs, antigen-presenting and innate immune DCs, Mφs, and barrier/stromal units (ECs and FBs)—we delineate their tissue-contextual coupling, construct a cross-disease metabolism–signaling network map, and discuss multidimensional translational strategies and companion diagnostics spanning pharmacologic, nutritional, exercise-based, and microbiota-focused interventions. We advocate the concept of “metabolically precise allergology”, envisioning that single-cell and spatial metabolomics integrated with artificial intelligence models will chart individual “metabolic–immune fingerprints”, enable stratified interventions and dynamic monitoring, and shift allergic disease management from passive inflammation control to proactive restoration of homeostasis in the era of precision medicine.

## Metabolic Reprogramming in Type 2 Inflammatory Disease

Systemic reprogramming of cellular metabolic networks represents a core feature of the disrupted coupling between immune and metabolic homeostasis. During antigen stimulation and inflammation maintenance, immune effector cells dynamically modulate energy metabolism pathways to achieve functional shaping; the choice of metabolic routes and the variation of flux directly determine the intensity and persistence of inflammatory responses [12]. Within this framework, alterations in glucose metabolism, fatty acid metabolism, amino acid metabolism, and global metabolic homeostasis constitute 4 key metabolic dimensions of allergic inflammation.

Rewiring of glucose metabolism provides both energetic and signaling support for effector cell activation and inflammatory amplification. Fatty acid metabolism contributes dual functions—energy supply and signaling modulation—participating in both initiation and resolution of inflammation. Amino acid metabolism regulates immune thresholds through nutrient sensing and metabolic flux control, whereas systemic metabolic transitions shape immune cell functional lineages and inflammatory phenotypes at the organismal level. Collectively, type 2 inflammatory diseases can be viewed as “metabolism-driven immune remodeling” processes, in which the pathological traits originate from the tight coupling of energy metabolism, signal transduction, and phenotypic expression [13].

Revisiting the pathogenesis of type 2 inflammatory diseases from a metabolic perspective not only elucidates their molecular underpinnings but also provides a theoretical foundation for precision interventions centered on metabolic regulation. This viewpoint lays the groundwork for in-depth exploration of the mechanistic associations among carbohydrate, fatty acid, amino acid, and systemic metabolic states and inflammatory phenotypes (Table 1).

**Table 1.** Metabolic orchestration of immune cell fate and function in type 2 inflammatory diseases

Metabolic type	Cells/tissues	Direction of change	Function/mechanism	Impact on inflammatory phenotype
Glucose metabolism	Th2, ILC2s, MCs, Eos	↑ Glycolysis/oxidative imbalance	Provides rapid ATP and metabolic intermediates; amplifies inflammatory signaling	Drives acute and chronic allergic responses and sustained inflammation
NECs, ILC2s	↑	Enhanced glucose uptake → lactate export	Amplifies type II inflammation; maintains a chronic inflammatory microenvironment
NECs, ILC2s	↑	Promotes glycolytic flux	Supports proinflammatory output
NECs, ILC2s	↑	Promotes lactate generation and export	GPR81/histone lactylation regulate inflammatory genes
Th2, ILC2s	Activated	Amplifies inflammatory signaling and glycolysis	Forms a bidirectional metabolism–immunity amplification loop
Th2, ILC2s	↑	Selectively promotes IL-5 and IL-13 transcription	Maintains Th2/ILC2 activation and tissue remodeling
MCs	Dependent	Drives degranulation under IL-33 or IgE stimulation	Promotes release of proinflammatory mediators
ECs, ILC2s, Th2	↑	“Back-education” of effector cells, sustaining chronic inflammatory milieu	Increases type II inflammatory burden
Multiple cell types	-	Links FA synthesis/redox balance, coupling glucose and lipid metabolism	Influences cross-regulation of immunometabolism
Airway local tissues	↑	Increases FcεRI and other receptor-signal sensitivity	Promotes inflammation and hyperreactivity
Fatty acid metabolism	Airway ILC2s	↑	Enhances FA uptake and FAO, supporting proliferation and IL-5/IL-13 secretion	Exacerbates ILC2-dependent airway inflammation
Activated ILC2s	Shift toward FAO	Greater FAO dependence under type II inflammation	Provides rationale for lipid-targeted therapy
MCs, ILC2s, Eos, Th2	Recruit/activate ILC2s; induce migration and IL-5/IL-13 release	Amplifies eosinophilic inflammation and bronchial hyperreactivity	COX–HPGDS–PGD_2_ inhibition; CRTH2 antagonism; CysLT receptor blockade
Neutrophils, Mφs, ECs	-	Limit neutrophil infiltration; promote Mφ clearance and repair	Improve inflammation and airway function (n-3 PUFAs supplementation)
Maternal plasma/placenta and infant airways	May reduce offspring wheeze/asthma risk	Potential preventive effect (n-3 LC-PUFA dietary intervention)	-
ECs, Mφs, ILC2s	-	Inhibit STAT6 and Akt pathways	Mitigate allergic inflammation and remodeling (receptor agonists)
Bronchial tissues	-	FFA levels and receptor activation linked to airway inflammation	Intervenable association
Pulmonary ILC2s	-	Regulates intracellular lipid partitioning; restrains ILC2-mediated inflammation	Reduces allergic lung inflammation
Adipose tissue; pulmonary/airway ILC2s	-	Systemic metabolic status remotely modulates type II inflammatory intensity	Shapes programming of allergic inflammation
Amino acid metabolism	ILC2s, Th2	↑	Leu uptake activates mTORC1, amplifying IL-5/IL-13 and clonal expansion	Enhances type II inflammatory output
Airway tissues	Arg1/Arg2 ↑	Competitive Arg consumption limits NO generation; polyamine/proline branches promote remodeling	Associated with asthma severity and remodeling
Skin barrier/AD axis	Altered/decreased	Microbial indoles activate AhR, strengthen barrier, and suppress type II inflammation and pruritus	Alleviates skin inflammation; promotes repair
Metabolic status and phenotype	Mφs	Differentiation-dependent	Metabolic pathways determine effector functions and fate	Explains differences between acute and chronic responses
Th2	Dependent	High-secretory phase relies on glycolysis; finely tuned by mTORC1	mTORC1 inhibition reduces effector output
ILC2s	Reprogrammed	Meets energy needs for proliferation/hypersecretion; sensitive to mTOR and ROS scavenging	Displays high metabolic plasticity
Asthma/upper-airway mucosa	Increased	Associated with eosinophilic infiltration, goblet-cell metaplasia, mucus hypersecretion, airway hyperreactivity	Inhibition of this axis alleviates inflammation and hyperreactivity
Multiple tissues/cells	Context-dependent	Can enhance proinflammatory responses or induce immunosuppression/repair depending on context	Determines amplitude and persistence of type II inflammation

### Glucose metabolism

Glucose metabolism has been established as a central hub of immunometabolic remodeling in type 2 inflammatory diseases, with alterations spanning the activation, functional maintenance, and persistence of immune effector cells [7]. Growing evidence indicates that allergic reactions are not merely immune abnormalities but rather results of systemic reprogramming within cellular metabolic networks. In both acute allergic responses and chronic hypersensitivity inflammation, Th2 cells, ILC2s, MCs, and Eos exhibit markedly enhanced glycolysis and disrupted mitochondrial oxidative metabolism [14]. This “Warburg-like” metabolic shift provides rapid adenosine triphosphate (ATP) and metabolic intermediates while amplifying inflammatory signaling via the HIF-1α–mTOR–PFKFB3/pyruvate dehydrogenase kinase 1 (PDK1) axis, forming a bidirectional feedforward loop between metabolism and immune activation.

In asthma and AR, nasal epithelial cells (NECs) and ILC2s show up-regulated expression of glucose transporter 1 (GLUT1), hexokinase 2 (HK2), and lactate dehydrogenase A (LDHA), promoting glucose uptake and lactate efflux [15]. Lactate acts not only as a metabolic by-product but also as a signaling molecule that engages the lactate receptor GPR81 and induces histone lactylation, thereby regulating the epigenetic transcriptional landscape of inflammation-related genes [16]. Lactylation selectively enhances transcription of IL-5 and IL-13, sustaining Th2 and ILC2 activation and tissue remodeling. Furthermore, MCs rely on glycolysis-derived ATP and NADPH (reduced form of nicotinamide adenine dinucleotide phosphate) for degranulation under IL-33 or IgE stimulation, whereas inhibition of glycolysis with 2-deoxy-d-glucose (2-DG) markedly suppresses their proinflammatory mediator release [17]. Together, these findings establish glucose metabolism as not only a source of bioenergetic substrates but also a master regulator of signaling and inflammatory phenotypes.

Emerging evidence suggests that glucose metabolism exerts a “third level” role in integrating immune–epithelial interactions. Abnormal activation of glucose metabolism in airway ECs enhances intrinsic inflammatory responses while releasing IL-33, TSLP, and lactate as metabolic–inflammatory mediators that “educate back” ILC2s and Th2 cells, thereby maintaining a chronic inflammatory milieu [18]. In addition, glycolytic intermediates such as pyruvate can enter mitochondria to participate in fatty acid synthesis and redox homeostasis, metabolically linking carbohydrate and lipid metabolism to achieve cross-regulation [19]. Notably, in chronic allergic airway inflammation, a local hyperglycemic environment can promote glycosylation and enhance IgE receptor sensitivity, indicating that glucose may modulate immune receptor function via metabolic posttranslational modification [20]. Therapeutic strategies targeting glucose metabolism have shown translational promise. Pharmacologic inhibition of HIF-1α or PFKFB3 by PX-478 or 3-(3-pyridinyl)-1-(4-pyridinyl)-2-propen-1-one (3PO) substantially reduces airway hyperresponsiveness and mucus hypersecretion in asthma models [21]. Similarly, the lactate dehydrogenase inhibitor GNE-140 and GLUT1 inhibitor BAY-876 selectively attenuate the proinflammatory activity of ILC2s without affecting their survival [22]. These findings highlight metabolic intervention as a potential therapeutic avenue for type 2 inflammatory diseases.

### Fatty acid metabolism

Fatty acid metabolism constitutes another critical hub in the metabolic reprogramming underlying allergic inflammation. On the one hand, fatty acid uptake and β-oxidation provide energy and epigenetic substrates for 2-type effector cells such as ILC2s and Th2 cells; on the other hand, lipid mediators—including eicosanoids and specialized pro-resolving mediators (SPMs)—translate metabolic cues into inflammatory or pro-resolving signals, jointly constructing a “metabolism–signaling” coupling network within airway and mucosal barriers.

At the cellular level, IL-33 induces ILC2s to up-regulate peroxisome proliferator-activated receptor γ (PPARγ), which enhances CD36-mediated fatty acid uptake and promotes FAO, thereby supporting ILC2 proliferation and IL-5/IL-13 secretion. Pharmacological antagonism or genetic ablation of PPARγ or its downstream lipid metabolic program attenuates fatty acid utilization and energy supply, substantially reducing ILC2-dependent airway inflammation [23]. Notably, activated ILC2s can utilize both glucose and fatty acid substrates, but within type II inflammatory contexts, their reliance on FAO becomes predominant—providing a rationale for therapeutic strategies targeting lipid metabolism to suppress ILC2 activation [24].

Lipid mediators translate fatty acids into inflammatory “instructions”. During IgE- or FcεRI-triggered immediate responses, MCs rapidly produce prostaglandin D₂ (PGD₂) and cysteinyl leukotriene (CysLT), notably leukotriene E₄ (LTE₄). These act through CRTH2 [also known as d-prostanoid receptor 2 (DP2)] and CysLT receptors to recruit and activate human ILC2s, inducing migration and IL-5/IL-13 release that amplify eosinophilic inflammation and bronchial hyperreactivity [25]. Blocking the cyclooxygenase (COX)–hematopoietic prostaglandin D synthase (HPGDS)–PGD₂ axis or antagonizing CRTH2 suppresses ILC2 activation; similarly, CysLT receptor blockade mitigates ILC2 proinflammatory phenotypes and type II inflammatory burden [26], supporting the mechanistic foundation for CRTH2 antagonists and antileukotriene therapy in allergic airway diseases.

Beyond proinflammatory lipids, pro-resolving lipid mediators are essential for restoring inflammatory homeostasis. n-3 polyunsaturated fatty acid-derived SPMs—including resolvins, protectins, and maresins—limit neutrophil infiltration, enhance Mφ clearance, and promote tissue repair, thereby ameliorating inflammation and airway dysfunction in respiratory disease models [27]. Thus, the host fatty acid profile determines not only inflammatory magnitude but also outcome, guiding resolution through SPM biosynthesis.

Host fatty acid supply and sensing pathways can also reshape allergic susceptibility. Relationships between prenatal or early-life exposure to n-3 LC-PUFAs and reduced offspring wheeze or asthma risk have been evaluated in multiple randomized controlled trials and meta-analyses. Although the overall trend supports a protective effect of LC-PUFA supplementation during pregnancy, the conclusions remain influenced by dose, timing, and outcome heterogeneity [28]. At the signaling level, GPR120 and free fatty acid receptor 4 (FFAR4) exert anti-inflammatory effects in airway ECs and immune cells. Agonism of these receptors inhibits signal transducer and activator of transcription 6 (STAT6) and protein kinase B (Akt) pathways, attenuating allergic inflammation and airway remodeling. Both experimental and clinical studies of obesity-related asthma further support an intervention-sensitive link among circulating free fatty acid (FFA) levels, receptor activation, and airway inflammation [29].

In addition, fatty acid-binding protein 5 (FABP5) restricts ILC2-mediated allergic pulmonary inflammation, underscoring intracellular lipid transport and metabolic partitioning as “hidden regulators” of type 2 response intensity [30]. Meanwhile, the adipose tissue–ILC2 axis and dietary saturated fatty acid uptake reveal that systemic metabolic status exerts distal control over the programming of allergic inflammation.

### Amino acid metabolism

In type 2 inflammatory diseases, effector lymphocytes undergo systemic reprogramming in amino acid uptake, sensing, and flux, which directly determines the intensity and persistence of type II inflammatory responses. ILC2s and Th2 cells in mucosal barriers are particularly sensitive to amino acid availability, with leucine (Leu) uptake and its downstream signaling acting as critical metabolic gatekeepers. ILC2s up-regulate transporters such as the L-type amino acid transporter 1 (LAT1) to enhance Leu import, thereby activating mTORC1 as a nutrient-sensing hub that amplifies IL-5 and IL-13 production and clonal expansion capacity. Studies have shown that amino acid supply itself constitutes a “metabolic throttle” for ILC2 responses; reducing amino acid availability or inhibiting mTOR signaling markedly decreases type II inflammatory output [12].

In airway inflammation, the Arg–nitric oxide (NO)–arginase (ARG) axis forms a classical amino acid metabolic node. Up-regulation of Arg1 and Arg2 in asthmatic airways competitively depletes Arg, restricting NO synthesis and promoting airway hyperreactivity, while branching into polyamine and proline pathways that contribute to smooth muscle hypertrophy, mucus secretion, and airway remodeling. Reduced Arg bioavailability and elevated ARG activity correlate strongly with asthma severity and remodeling extent [31]. Intervention studies demonstrate that Arg supplementation or ARG modulation can improve inflammatory and physiological phenotypes, although clinical efficacy depends on subtype stratification and optimized combination regimens.

Along the skin–allergy axis, the Trp–kynurenine/indole–AhR pathway integrates both metabolic and signaling functions. Patients with AD show significant abnormalities in Trp metabolic profiles, whereas indole metabolites produced by commensal skin microbiota activate AhR, enhancing skin-barrier function while suppressing type II inflammation and pruritus pathways. Exogenous or microbiota-derived Trp metabolites alleviate skin inflammation and promote barrier repair in animal models, suggesting that the Trp–AhR axis may serve as a metabolic–microbial target regulating skin–airway inflammatory crosstalk [32].

These findings provide mechanistic rationale and translational potential for targeting amino acid metabolism: (a) inhibiting LAT1 or down-regulating mTORC1 activity lowers the metabolic threshold and inflammatory peak of ILC2s and Th2 cells, as exemplified by the LAT1 inhibitor JPH-203; (b) restoring the Arg–NO dynamic balance may alleviate airway hyperreactivity and remodeling; (c) leveraging the Trp–AhR axis via host–microbiota interactions can strengthen EC barriers and suppress type II inflammation [3,5].

### Metabolic state and inflammatory phenotype

During immune cell functional shaping, metabolic state serves as a decisive coordinate. Classically, proinflammatory M1-type Mφs are characterized by reinforced glycolysis and lactate generation, accompanied by HIF-1α stabilization and activation of the pentose phosphate pathway (PPP); conversely, anti-inflammatory M2-type Mφs rely on FAO and mitochondrial OXPHOS to support tissue repair and immune regulation. This metabolic–phenotypic dichotomy provides a basic framework for explaining functional differences in acute and chronic inflammation and has been validated in multiple animal models and human samples [33]. These data indicate that metabolic pathways are not only energetic engines but also phenotypic switches that determine cell fate and effector functions.

Notably, such metabolism-driven phenotypic shaping is not restricted to myeloid cells but represents a cross-lineage mechanism. During differentiation and high-level secretion of IL-4, IL-5, and IL-13, Th2 cells display marked glycolytic dependence under fine control of mTORC1; mTORC1 inhibition reduces metabolic flux and weakens effector output [34]. Similarly, ILC2s rely mainly on OXPHOS at steady state but rapidly undergo metabolic reprogramming under potent stimuli such as IL-33. Glycolytic enhancement, accumulation of reactive oxygen species (ROS), and coordinated up-regulation of lipid metabolism and mitochondrial flux collectively meet the bioenergetic needs of proliferation and cytokine hypersecretion [35]; this process is sensitive to mTOR activity and can be attenuated by rapamycin or ROS scavengers, indicating high metabolic plasticity [36].

This cross-lineage metabolic remodeling has direct pathological relevance in type 2 inflammatory diseases. In asthma and upper-airway allergic mucosa, enhanced glycolysis and lactate accumulation correlate closely with activation of signaling axes including pyruvate kinase M2 (PKM2) and HIF-1α, and are positively associated with type II inflammatory features such as eosinophilic infiltration, goblet-cell metaplasia, mucus hypersecretion, airway hyperreactivity, and disease activity [37]. Inhibition of the HIF-1α–glycolysis–lactate signaling axis significantly alleviates airway inflammation and hyperreactivity in experimental models, providing causal support for the “metabolism–phenotype” mapping logic. From a “nose–bronchial unity” perspective, differences in metabolic dependency across barrier tissues such as airways and skin may also explain organ-specific clinical phenotypes and therapeutic responses [38].

**Fig. 1.**
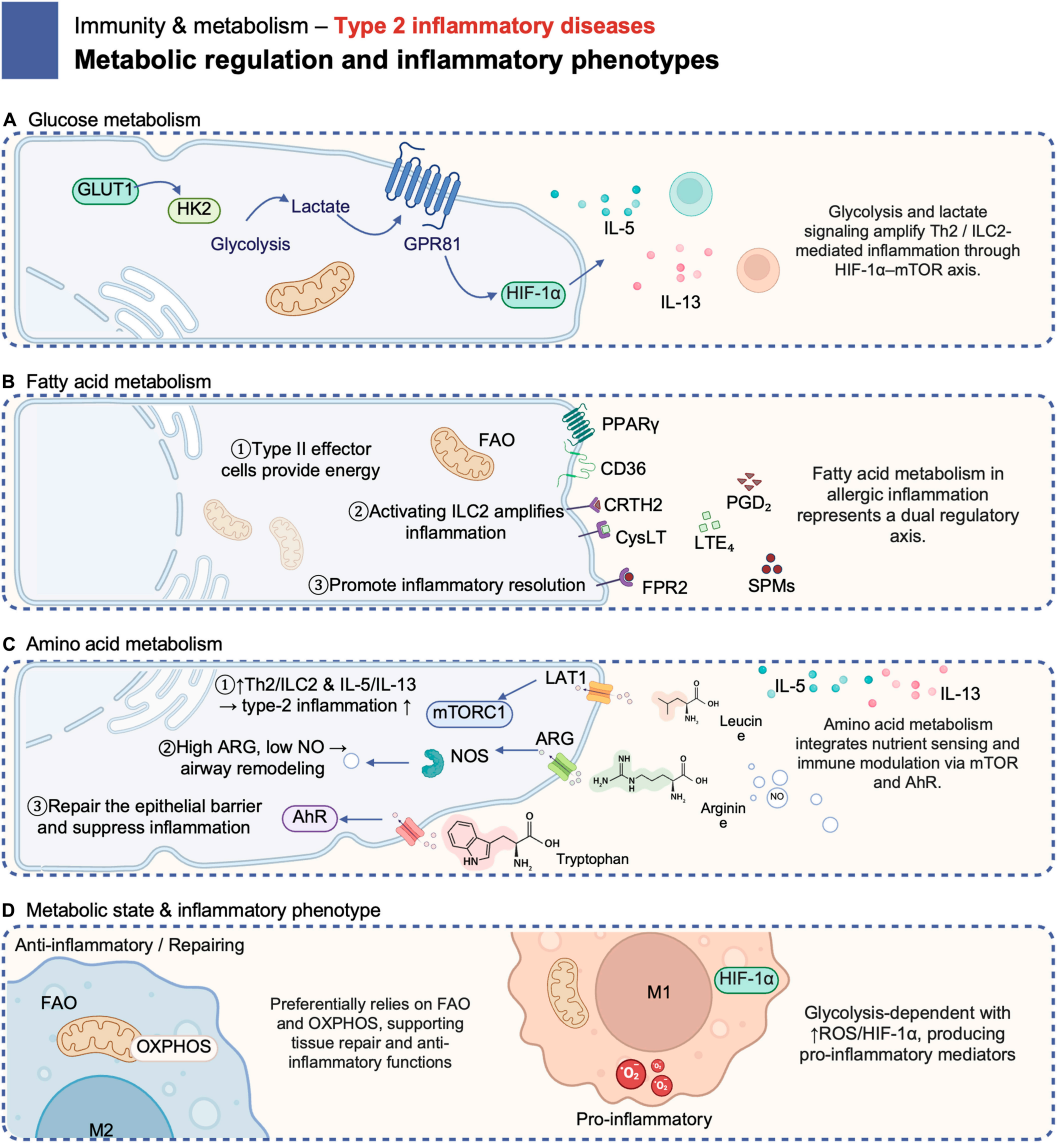
Metabolic regulation and inflammatory phenotypes in type 2 inflammatory diseases. (A) Glucose metabolism: Th2 cells and ILC2s rely on glycolysis; lactate signals through GPR81 and the HIF-1α–mTOR axis to amplify IL-5/IL-13-mediated inflammation and contribute to epithelial repair. (B) Fatty acid metabolism: FAO fuels effector cells; PGD₂ and cysteinyl-leukotrienes (cysLTs) acting via CRTH2 and related receptors enhance inflammation, whereas specialized pro-resolving mediators (SPMs) acting through FPR2 promote resolution; PPARγ and CD36 participate in lipid-immune regulation. (C) Amino acid metabolism: LAT1→mTORC1 links nutrient sensing to immune activation; the arginine (ARG)/nitric oxide (NO) balance shapes airway remodeling; tryptophan metabolites signal through AhR to maintain the epithelial barrier and suppress inflammation. (D) Metabolic state and inflammatory phenotype: M2 macrophages (Mφs) favor FAO and mitochondrial oxidative phosphorylation (OXPHOS), supporting tissue repair and anti-inflammatory functions; M1 Mφs are glycolysis-dependent with elevated reactive oxygen species (ROS) and HIF-1α, producing pro-inflammatory mediators.

Metabolites themselves possess signaling functions. Lactate can be shuttled between cells via monocarboxylate transporter 1 (MCT1) and monocarboxylate transporter 4 (MCT4), or signal through GPR81. Depending on context, these mechanisms may either potentiate proinflammatory responses or induce immunosuppression and tissue repair. This “metabolism–receptor axis” reveals the molecular basis by which the same metabolic alteration can lead to opposite immune outcomes across tissues or disease stages [39]. Consequently, the microenvironment-defined metabolic landscape—including oxygen tension, substrate availability, and lactate export/recycling capacity—determines the amplitude and persistence of type II inflammatory responses, thereby shaping the onset, chronicity, and recurrence of type 2 inflammatory diseases [19]. Related studies show that maternal glycemic status during pregnancy is linked to allergic risk in the mother–infant dyad [40]. Maternal diabetes—including gestational diabetes mellitus (GDM)—is associated with higher risks of childhood allergic diseases and asthma, supporting a prenatal metabolic programming effect on type 2 immunity [41,42]. Related studies also show that higher maternal free-sugar intake during pregnancy is associated with increased atopy and AS at school age, independent of the child’s own sugar intake, implicating prenatal glucose exposure as an upstream driver of allergic sensitization [43]. Mechanistically, in utero hyperglycemia may influence fetal immune and epithelial development—via mTOR/HIF-1α–glycolysis signaling, redox tone, and microbiota-derived metabolites—biasing Th2 responses and barrier maturation; consequently, maternal glucose metabolism emerges as a modifiable determinant of allergic susceptibility across generations [44] (Fig. 1).

## Metabolic Remodeling of Allergic Effector Cells

The persistence of inflammation and tissue remodeling in type 2 inflammatory diseases arises from dynamic metabolic reprogramming across multiple immune cell types and their mutual amplification. T cells occupy a central position: Pathogenic Th2 (*pTh2*) cells and tissue-resident memory Th2 cells (*Th2-TRM*) rely primarily on FAO at rest to maintain energetic homeostasis but rapidly switch to a high-throughput, glycolysis-dominant mode upon exposure to allergens, IL-33, or the epithelial alarmin TSLP, thereby driving abundant secretion of IL-4, IL-5, and IL-13 and sustaining local inflammation [45]. At the humoral level, B cells and PCs sustain pathogenic IgE through memory clones and long-lived PCs. The T follicular helper type 13 (*Tfh13*) subset promotes high-affinity IgE via high IL-13 signaling and is considered a key driver of severe allergic reactions [46].

ILC2s act as early amplifiers at barrier tissues and are pivotal in asthma and severe eosinophilic phenotypes. Highly sensitive to epithelial cytokines such as IL-33, IL-25, and TSLP, ILC2s display enhanced glycolysis with coordinated up-regulation of FAO, forming a positive feedback loop between metabolism and inflammation. Steroid-insensitive ILC2 subsets exhibit stronger metabolic activity, correlating positively with clinical severity [47]. Clinically, blockade of the epithelial–ILC2 axis with biologics, such as the anti-TSLP agent tezepelumab, reduces inflammatory burden in asthma with or without nasal polyps, further supporting the core role of metabolic–immune synergy in amplifying allergic inflammation [48,49].

Eos are key effector cells in allergic inflammation and exhibit pronounced metabolic plasticity in tissues. They can flexibly switch between glycolysis and FAO to sustain survival and mediator release. The metabolic adaptability of Eos is closely related to tissue persistence and proinflammatory intensity and may influence differential responses to anti-IL-5 or IL-5 receptor (IL-5R) therapies. Beyond the classic IgE–high-affinity FcεRI pathway, MCs also sense neuropeptides and small molecules through G protein-coupled receptors (GPCRs) such as MRGPRX2, triggering Ca^2+^-dependent degranulation and inflammatory signaling; their metabolic state may, together with receptor thresholds, determine the degree of pruritus and vascular leakage [50].

DCs couple antigen presentation with metabolic control: Glycolysis-biased DCs tend to enhance costimulation and Th2 polarization, whereas FAO/OXPHOS-dominant DCs more readily acquire tolerogenic phenotypes, providing a metabolic basis for peripheral tolerance [51]. Metabolic polarization of Mφs likewise directs inflammation, with glycolysis-dominant Mφs being proinflammatory and FAO/OXPHOS-dominant Mφs contributing to anti-inflammatory functions and tissue repair [52].

**Fig. 2.**
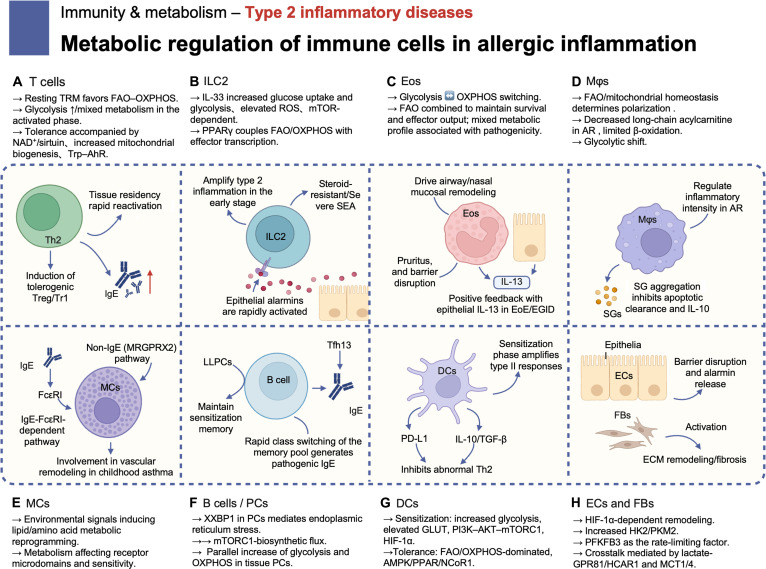
Metabolic regulation of immune cells in allergic inflammation. (A) T cells: Quiescent tissue-resident memory T (TRM) cells rely on fatty acid oxidation (FAO) and oxidative phosphorylation (OXPHOS); activation shifts toward glycolysis. Tolerance involves nicotinamide adenine dinucleotide (NAD^+^)/sirtuin signaling, mitochondrial biogenesis, and the tryptophan (Trp)–aryl hydrocarbon receptor (AhR) pathway. (B) ILC2s: Interleukin-33 (IL-33) increases glucose uptake and glycolysis with ROS and mechanistic target of rapamycin (mTOR) activation; peroxisome proliferator-activated receptor-γ (PPARγ) couples FAO/OXPHOS to effector programs. (C) Eosinophils (Eos): Switching between glycolysis and OXPHOS sustains survival and effector output; FAO-driven hybrid metabolism promotes mucosal remodeling and interleukin-13 (IL-13) feedback. (D) Macrophages (Mφs): FAO and mitochondrial integrity shape polarization; glycolytic shift and stress granule (SG) formation tune inflammation and fibrosis. (E) Mast cells (MCs): Lipid/amino acid cues drive metabolic reprogramming, modulating FcεRI-dependent and FcεRI-independent activation and vascular remodeling in pediatric asthma. (F) B cells/plasma cells (PCs): X-box binding protein 1 (XBP1)-mediated endoplasmic reticulum (ER) stress and mTOR complex 1 (mTORC1)-linked biosynthesis enhance glycolysis/OXPHOS to sustain antibody secretion. (G) Dendritic cells (DCs): Sensitization up-regulates glycolysis via phosphoinositide 3-kinase (PI3K)–AKT–mTORC1 and HIF-1α; tolerance relies on FAO/OXPHOS via AMP-activated protein kinase (AMPK)/PPAR/nuclear receptor corepressor 1 (NCoR1). (H) Epithelium/fibroblasts (FBs): HIF-1α-driven remodeling elevates hexokinase 2 (HK2)/pyruvate kinase M2 (PKM2); lactate signals via G protein-coupled receptor 81 (GPR81)/hydroxycarboxylic acid receptor 1 (HCAR1) and monocarboxylate transporters 1/4 (MCT1/4) to promote inflammation and fibrosis.

At the tissue level, ECs and FBs form a cooperative ring of metabolism–inflammation–remodeling. In airway and nasal mucosa, glycolytic shift and lactate accumulation under allergic inflammation and hypoxia activate FBs via GPR81, inducing α-SMA and collagen synthesis and driving fibrosis and structural remodeling [53]. Overall, the onset and chronicity of type 2 inflammatory diseases reflect a multicellular, multilayered metabolic coordination network: Metabolic activation of T cells and ILC2s, energetic adaptation of Eos and MCs, secretory load of B cells and PCs, metabolic polarization of DCs and Mφs, and the epithelial–stromal glycolysis–lactate feedback collectively constitute the energy and signaling engine of allergic inflammation (Fig. 2).

### Adaptive immunity

#### T cells: The metabolic–epigenetic axis in immune memory

T cells are central to the initiation, maintenance, and rebuilding of immune tolerance in type 2 inflammatory diseases. Their functional diversity and tissue distribution largely shape phenotypes and disease course. This centrality is sculpted not only by cytokine networks but also by a multilayered “nutrient uptake–metabolic sensing–epigenetics” axis.

In asthma, recent studies delineate *pTh2* and *Th2-TRM* populations that persist long-term within airway mucosa, possess local clonal expansion and rapid reactivation capacity, and can swiftly secrete large amounts of IL-4, IL-5, and IL-13 to drive chronic airway hyperreactivity and structural remodeling. Metabolically, these cells favor FAO and OXPHOS during tissue residency to maintain mitochondrial fitness and survival, but upon inhaled allergens or epithelial “alarmin” stimulation rapidly switch to glycolysis or mixed metabolism. Through LAT1–mTORC1, PFKFB3–HIF-1α, and PDK1, and by limiting pyruvate entry into the tricarboxylic acid cycle (TCA), they elevate ATP and biosynthetic flux to sustain high-level cytokine production and proliferation [54,55]. Single-cell transcriptomics and chromatin accessibility further suggest coupling between metabolism and chromatin-histone acetylation via acetyl-coenzyme A (CoA), and one-carbon metabolism control of methyl donors—aligned with pathogenic programs, providing a molecular basis for relapse and differential responses to anti-IL-4Rα and anti-IL-5 biologics [56].

In skin disease, AD features both systemic Th2 responses and intradermal CD4^+^ TRM with metabolic–transcriptional specialization. These TRM tend to use OXPHOS, lipid droplet (LD) mobilization, and Gln catabolism to maintain low-grade yet persistent activity and survival. When neuro-immune signals or microbial metabolites (indoles, lactate) rise, thresholds of metabolic sensors such as mTOR, AMPK, and AhR can be reset, inducing locus-specific activation of chemokine and barrier-repair genes [57,58]. Multi-omics and spatial transcriptomics have resolved TRM subsets linked to barrier repair, chemotaxis, and local immune balance, suggesting that “local metabolic–immune memory” may underlie the difficulty in achieving durable tolerance and the propensity for relapse in AD. Intriguingly, a parallel metabolic–epigenetic memory is observed in alopecia areata (AA), where hair bulb-resident CD8^+^ T cells undergo a hyper-glycolytic shift to sustain chronic interferon-γ (IFN-γ) production, locked in a state that drives recurrent hair follicle destruction [59,60].

In FA, T cell dynamics between sensitization and tolerance highlight the plasticity of metabolic thresholds. Allergic individuals often harbor low-frequency yet highly pathogenic allergen-specific CD4^+^ memory subsets (*Th2A* and pathogenic Th2) in gut mucosa and peripheral blood; these depend on LAT1–mTORC1–c-Myc and glycolysis–nucleotide synthesis programs to promote B cell IgE class switching and amplify immediate responses of MCs and Eos [61]. Conversely, allergen-specific immunotherapy (AIT) or oral immunotherapy (OIT) tends to induce more oxidative Tregs and Tr1 while down-regulating glycolytic features in effector memory T cells; with remodeling of the nicotinamide adenine dinucleotide (NAD^+^)–deacetylase family–mitochondrial biogenesis axis and the Trp–AhR pathway, tolerance-associated epigenetic states are stabilized.

As metabolic and functional antagonists to pathogenic Th2 cells, Tregs are indispensable for maintaining immune rheostasis. At steady state, Tregs preferentially utilize FAO and mitochondrial OXPHOS to sustain their suppressive capacity and lineage stability, a program coordinated by the AMPK and liver kinase B1 (LKB1) signaling axis [62]. However, in the nutrient-dense and inflammatory microenvironment of type 2 diseases, Tregs exhibit significant metabolic and functional plasticity. Overactivation of the glycolysis–mTORC1 pathway can destabilize the FOXP3 transcriptional program, causing Tregs to lose their suppressive function and switch toward a pro-inflammatory, Th2-like phenotype (termed “Ex-Tregs”) [63]. This metabolic-driven “tolerance collapse” contributes to the persistence of inflammation in conditions like EoE and chronic asthma. Conversely, successful therapeutic interventions, such as AIT, aim to restore the metabolic fitness of Tregs—re-engaging the Trp–AhR and NAD^+^–sirtuin pathways to stabilize tolerance-associated epigenetic states and suppress aberrant type 2 responses.

#### B cells and PCs: Metabolic niches for IgE production and survival

The roles of B cells in type 2 inflammatory diseases extend far beyond “antibody factories”. Subset heterogeneity, memory formation and immunoregulation follow differentiated, clinically relevant trajectories across diseases and stages.

In FA, recent human studies identify type 2-polarized memory B cells (*MBC2*) and *CD23^+^IgG1^+^* memory B cells as enriched in allergic individuals; they carry high-affinity allergen-specific clones and rapidly class-switch to pathogenic IgE upon re-exposure, providing a cellular basis for relapse and long-term susceptibility. Two complementary studies show that *MBC2* serve as a key “reservoir” for pathogenic IgE, while *CD23^+^IgG1^+^* memory cells transcribe immunoglobulin heavy constant epsilon (*IGHE*), associate with serum IgE levels, and give rise to high-affinity IgE-producing cells [64,65]. This process critically depends on T follicular helper–germinal center (Tfh-GC) dynamics. *Tfh13* is required for “high- rather than low-affinity” IgE production; its “high IL-13 with relatively limited IL-21” signaling biases GC reactions toward IgE class switching and affinity maturation and correlates with higher risk of anaphylaxis [66]. Notably, in patients with severe anaphylaxis, specific IgE^+^ PC subsets exhibit highly active metabolic states to support the rapid and massive synthesis of IgE antibodies; this high-velocity secretion requires precisely coordinated energy flux and metabolic adaptations within the secretory niche.

In asthma, changes in circulating and local B cell compartments—such as increased *CD27^+^CD38^+^* plasmablasts and PCs, enrichment of antigen-specific memory B cells, and associations with serum IgE and severity—suggest that B cell dynamics may serve as biomarkers for stratification and response monitoring [67,68]. Functionally, beyond activating MCs and Eos via the IgE axis, context-dependent immunoregulation by B cells is notable. B cell-derived IL-10 can, in early sensitization, promote DC recruitment and Th2 initiation via epithelial–chemokine axes, but in late responses or under AIT tends to be anti-inflammatory and tolerance-promoting; induction and maintenance of regulatory B cells (Bregs) are considered important mechanisms of AIT benefit [67,69].

As terminally differentiated progeny, PCs are pivotal to initiation, maintenance, and relapse of allergic inflammation. Long-lived plasma cells (LLPCs) establish tissue niches and, dependent on survival signals from stress granules (SCs), DCs, and FBs, continuously secrete antigen-specific IgE and IgG1 to maintain sensitization memory; their high-load secretion relies on X-box binding protein 1 (XBP1)-mediated ER stress programs and mTORC1-driven biosynthetic flux [70,71]. This metabolic commitment is essential for maintaining the persistent pathogenic IgE levels seen in chronic allergy and life-threatening anaphylaxis.

CRSwNP, immunohistochemistry, and single-cell analyses show enrichment of IgE^+^ PCs coexpressing CD138, XBP1, and high B cell maturation antigen (BCMA); the B cell activating factor (BAFF)/a proliferation-inducing ligand (APRIL)–BCMA axis, together with epithelial- and FB-derived IL-6 and chemokine ligand 12 (CXCL12), sustains their survival and secretion, enabling continuous antibody synthesis even in hypoxic, high-lactate microenvironments—offering an explanation for relapse after surgery or systemic steroids [72,73].

In chronic asthma, airway PCs display residency and bidirectional crosstalk with epithelium and innate type II pathways: IL-33 and TSLP promote differentiation of B cells into IgE PCs, which in turn activate effector cells via FcεRI to form a local amplification loop. Metabolically, these tissue PCs show “parallel up-regulation of glycolysis and OXPHOS”; inhibition of PFKFB3 or mTORC1 markedly diminishes survival and secretion, providing druggable entry points for combined metabolism–immunity interventions [74,75].

### Innate immunity

#### ILC2s: Metabolic amplification of neuro-immune signaling

ILC2s reside at epithelial barriers and act as early amplifiers of allergic airway inflammation, particularly in severe eosinophilic asthma. Unlike adaptive immunity, ILC2s do not require antigen receptors; instead, they are rapidly activated by epithelial alarmins (IL-33, IL-25, TSLP) to secrete IL-5 and IL-13, thereby promoting Eos recruitment and activation, goblet-cell metaplasia, mucus production, and airway smooth muscle remodeling [76]. Human studies using bronchoalveolar lavage fluid (BALF) and sputum show that ILC2 frequency correlates positively with IL-5/IL-13 levels, airway hyperreactivity, and clinical severity, supporting their key role in maintaining chronic type II inflammation [77,78].

Under severe and inhaled corticosteroid (ICS)-resistant conditions, ILC2s display both “persistent activation” and “functional plasticity”. High expression of IL-33 and TSLP receptors in patient airways enables activation at low ligand levels, and positive feedback can form between ILC2s and Th2 cells. IL-13 produced by ILC2s promotes DC maturation and Th2 differentiation, whereas Th2-derived IL-4 and IL-9 further enhance ILC2 responses, forming a sustained amplification circuit of type II inflammation [79]. Single-cell transcriptomics in steroid-refractory cohorts has identified pathogenic ILC2 subsets with high IL-9 and amphiregulin (Areg), metabolic activity, reduced sensitivity to ICS, and tissue-resident memory-like features under persistent IL-33 signaling [80].

Metabolic reprogramming is a core intrinsic driver of ILC2 pathogenicity. IL-33 enhances glucose uptake and glycolysis in ILC2s, accompanied by increased ROS and mTOR-dependent amplification; inhibition of HIF-1α/glycolysis or intervention with rapamycin significantly reduces IL-13 production and alleviates airway inflammation [81]. In parallel, PPARγ couples FAO and OXPHOS to the transcriptional programs of ST2, IL-5, and IL-13; its inhibition attenuates allergic airway inflammation [82]. Metabolites modulate ILC2s through metabolism–receptor axes: SCFAs act via GPR43 and FFAR2 to suppress ILC2 function and reduce ILC2-dependent airway hyperreactivity, while lactate and certain lipid derivatives adjust ILC2 energy states and inflammatory thresholds through HCAR1, GPR81, and PPARγ [83].

Neuro–immune interactions further shape ILC2 activity. ILC2s selectively express neuromedin U receptor 1 (NMUR1), and its ligand neuromedin U (NMU) rapidly and potently activates ILC2s in vivo and ex vivo to amplify type II inflammation, linking the vagus nerve, gut–lung axis, and mucosal immunity, and providing physiological grounding for rhythm- and stress-related exacerbations [84,85].

Translationally, the epithelial–ILC2 axis is a major therapeutic target in severe asthma. Tezepelumab reduced exacerbations and improved lung function in phase 3 trials such as NAVIGATOR, with benefits across Eos strata and in patients with concomitant CRSwNP [48]; anti-IL-33 therapy itepekimab has shown phenotype-dependent results in late-stage respiratory trials, indicating the need for further precision stratification by immune and metabolic subtypes. Tissue-level enrichment of IL-33R^+^ ILC2s associates with relapse risk and holds potential as a predictor of response and recurrence.

Furthermore, the activity of the epithelial–ILC2 axis is significantly shaped by hormonal differences, contributing to the sex dimorphism observed in type 2 diseases [86]. Estrogen signaling has been shown to amplify ILC2-mediated airway inflammation and metabolic vigor, whereas androgens act as metabolic brakes on ILC2 proliferation and cytokine production, providing a mechanistic basis for the increased prevalence and severity of asthma in post-pubertal females [87].

#### Eos and basophils: Metabolic plasticity and tissue persistence

Eos are key effector cells of type II immunity with broad and heterogeneous pathogenic mechanisms across type 2 inflammatory diseases. Beyond classic mediators [major basic protein (MBP), eosinophil cationic protein (ECP), leukotrienes, and cytokines including IL-4, IL-5, and IL-13], recent evidence shows marked metabolic plasticity [88]. Eos can switch among glycolysis, aerobic glucose oxidation, and mitochondrial OXPHOS, and mobilize FAO in distinct microenvironments to support survival and effector output.

In eosinophilic asthma, Eos accumulate beneath the airway epithelium, promoting barrier disruption, goblet-cell metaplasia, and airway remodeling. Multi-omics indicates a mixed metabolic profile of glycolysis and FAO associated with enhanced inflammation and survival. Biologics targeting IL-5 or IL-5R markedly reduce Eos burden and improve lung function—for example, benralizumab (anti-IL-5Rα) reduces oral ICS dependence and exacerbations; mepolizumab (anti-IL-5) provides sustained benefit in severe asthma. However, variable efficacy suggests that simply lowering circulating or total numbers may not fully block tissue-level programs such as metabolic adaptation, survival, and localization [89].

In CRSwNP, Eos are enriched in nasal mucosa and polyps and associate with heightened IL-33 and transforming growth factor-β (TGF-β) signaling, fibrosis, and remodeling. Phase 3 studies show that mepolizumab and benralizumab reduce polyp size and nasal obstruction, with benralizumab achieving near depletion of Eos in blood and tissue [90]. Nevertheless, peripheral reductions do not always translate into tissue remission, suggesting “tissue-persistent” Eos subsets with self-maintenance, anti-apoptosis, and metabolic adaptation, now being delineated by spatial transcriptomics and single-cell metabolomics [91].

In skin and gastrointestinal disease, Eos also play central roles. In AD lesions, Eos contribute to pruritus and barrier damage via ECP and IL-31. Dupilumab (anti-IL-4Rα) significantly improves symptoms but is often accompanied by transient peripheral eosinophilia, possibly related to down-regulation of adhesion molecules such as vascular cell adhesion molecule-1 (VCAM-1) and cell redistribution [92]. In EoE and EGE, Eos form a positive feedback loop with epithelial IL-13, driving mucosal remodeling and barrier dysfunction. Epidemiology indicates rising incidence of EoE and high comorbidity with asthma and AR, reflecting a systemic type II immune background [93,94]. Notably, within the hypoxic microenvironment of the esophagus in EoE, Eos acts as a master metabolic switch; its stabilization enhances glycolytic flux and alters local energy metabolism, which in turn promotes Eos survival and amplifies tissue-specific inflammatory responses [95]. Furthermore, in eosinophilic granulomatosis with polyangiitis (EGPA), the metabolic remodeling of Eos—characterized by shifted nutrient utilization and heightened oxidative stress—serves as a primary driver of vascular endothelial damage and systemic vasculitis [96]. In DRESS, aberrant Eos expansion often relates to human leukocyte antigen (HLA)-restricted, drug-specific T cell receptor (TCR) activation and excessive IL-5, with an inflammatory pattern distinct from classic IgE-mediated allergy [97].

In eosinophilic chronic obstructive pulmonary disease (COPD), elevated peripheral Eos aligns with type II features, exacerbation risk, and responsiveness to inhaled ICS, and is considered a potential “treatable trait”. Transcriptomic and lipid-metabolic studies suggest up-regulation of cholesterol metabolism and the COX–prostaglandin E₂ (PGE₂) pathway, indicating that in nontypical type II contexts Eos can be reprogrammed toward pro-oxidative injury phenotypes—metabolic clues that inform stratified therapy [98].

Basophils (BASO) are rare but potent type 2 effector cells that participate in the initiation and amplification of allergic inflammation. Upon activation by IgE crosslinking, IL-33, or TSLP, they rapidly release histamine, leukotrienes, and Th2—promoting cytokines such as IL-4 and IL-13, thereby enhancing eosinophil recruitment and Th2/ILC2 polarization [99]. In asthma, BASO infiltration is associated with airway hyperreactivity and mucus production, while in AD, basophils cooperate with ILC2s and Eos to promote pruritus, epidermal inflammation, and barrier disruption. In CRSwNP, increased basophil numbers and IL-33 responsiveness have been linked to disease severity [100]. Emerging studies suggest that activated basophils undergo metabolic adaptation involving enhanced glycolysis and oxidative metabolism, which supports cytokine release and prolonged survival within inflamed tissues [101]. Collectively, BASO act as metabolically adaptable amplifiers of type 2 inflammation across multiple allergic diseases.

#### MCs: Immunometabolic imbalance and the plasticity of multimodal activation

The pathogenic core of MCs in type 2 inflammation is shifting from simple degranulation to an immunometabolic maladaptation, where dysregulated nutrient sensing and lipid fluxes perturb the balance between immune quiescence and hyperactivation.

In AD, cutaneous MCs, in addition to the classic IgE–FcεRI route, engage phosphoinositide 3-kinase (PI3K), phospholipase Cγ (PLCγ), mitogen-activated protein kinase (MAPK), notably extracellular signal-regulated kinase (ERK), and nuclear factor kappa-light-chain-enhancer of activated B cells (NF-κB) cascades to enhance degranulation and the synthesis of PGD₂ and leukotriene C_4_ (LTC₄). In non-IgE pathways, mas-related G protein-coupled receptor X (MRGPRX2) and mas-related G protein-coupled receptor B2 (MRGPRB2) sense neuropeptides, small-molecule drugs, and microbial products, and via Ca^2+^ influx through calcium release-activated calcium channel protein 1 (ORAI1) and ORAI2, together with ras homolog family member A (RhoA), rho-associated protein kinase (ROCK), and MAPK axes, trigger rapid degranulation and pruritus-related signaling. The natural product celastrol has been reported to inhibit MRGPRX2-related Ca^2+^ flux and degranulation in dermatitis models, reducing histamine and type II cytokines, suggesting druggability of the “MRGPRX2–CRAC (ORAI)” axis [102].

In CSU, MC activation is highly heterogeneous and serves as a primary driver of disease persistence. Beyond FcεRI crosslinking, recent evidence emphasizes that the activation of the MRGPRX2 pathway in the skin lesions of CSU patients is a central pathogenic hub. Notably, this heightened MRGPRX2 activity not only is a consequence of receptor overexpression but also is fundamentally linked to lipid-metabolic signaling abnormalities [103]. Dysregulated lipid pathways, particularly the reorganization of arachidonic acid and sphingolipid metabolism, can generate bioactive lipid mediators that retro-modulate the sensitivity and activation thresholds of MRGPRX2. Such variation in MRGPRX2 expression and function may enhance responses to substance P and compound 48/80, amplifying Ca^2+^ mobilization, ERK phosphorylation, and release of mediators such as IL-5 and tumor necrosis factor-α (TNF-α), and may relate to low or absent responses to the anti-IgE agent omalizumab in some patients [104]. The complement component 5a–complement receptor 5a (C5a-C5aR) pathway and the tissue factor–thrombin–protease-activated receptor (PAR) cascade can also directly or indirectly activate cutaneous MCs, inducing vasodilation and exudation consistent with wheals, erythema, and pruritus [105]. Circulating extracellular vesicles can promote MC activation via toll-like receptor 2 (TLR2) and TLR4–MAPK pathways, a phenomenon more prominent in antihistamine-refractory subgroups, highlighting “humoral triggers” in difficult-to-treat CSU [106].

In airway remodeling of childhood asthma, tryptase-family serine proteases released by MCs act through PAR-2 to affect endothelial–pericyte adhesion and stability, involving N-cadherin down-regulation and pericyte contraction, and associating with microvascular remodeling and increased leakage. Spatial transcriptomics and localization studies show up-regulated MC activation pathways in vessel-dense regions, implicating MCs in multicellular crosstalk among vasculature, stroma, and smooth muscle [107].

In AR models, MC metabolic phenotypes are also shaped by environmental cues. Ultra-high-performance liquid chromatography coupled with quadrupole time-of-flight mass spectrometry (UHPLC-QTOF-MS) metabolomics shows that upon lipopolysaccharide (LPS) or danger signals, beyond histamine and leukotrienes, arachidonic acid, histidine, and sphingolipid (SL) pathways are markedly reorganized; these lipid and amino acid metabolites not only enhance inflammation but also, by reshaping membrane lipids and receptor microdomains, may retro-modulate the sensitivity and thresholds of MRGPRX2 and FcεRI [108,109]. Overall, metabolism, ORAI/CRAC channels, and receptor layers (MRGPRX2, FcεRI, PAR, C5aR, TLR) form a plastic coupling network within MCs, sculpting their “pro-inflammation–pruritus–remodeling” spectrum across diseases and stages [108].

#### DCs: Metabolic gating of Th2 sensitization and tolerance

DCs serve as dual hubs—“antigen presentation–metabolic regulation”—across sensitization and maintenance. Following epithelial alarmins and allergen exposure, DCs rapidly increase glycolysis and glucose uptake with GLUT up-regulation; through PI3K–AKT–mTORC1 and HIF-1α-related axes, they boost ATP and biosynthetic substrates, up-regulate CD80/CD86, enhance C–C chemokine receptor type 7 (CCR7)-dependent migration, and amplify type II inflammation. Conversely, programs dominated by FAO and mitochondrial OXPHOS drive tolerogenic phenotypes (tolDCs) that, via PD-L1, IL-10, and TGF-β, restrain aberrant Th2 responses, in close association with metabolic sensors or transcriptional corepressors such as AMPK, PPAR, and nuclear receptor co-repressor 1 (NCoR1) [110].

In airway diseases such as asthma and CRSwNP, local hypoxia and lactate accumulation stabilize glycolytic programs via HIF-1α–mTORC1, inducing PDK1 and LDHA, and shift DC migration and polarization thresholds; lactate–HIF-1α may, via mitochondrial regulator NDUFA4L2, feedback-limit excessive inflammation, indicating a dynamic braking loop between “metabolism and inflammation” [19]. Single-cell and spatial transcriptomics in patient samples identify DC subsets with distinct metabolic fingerprints—some enriched for carbohydrate-metabolism genes and positively associated with IL-33/TSLP axes and disease severity, others up-regulating FAO, oxidative stress, and antigen-processing pathways. In eosinophilic versus noneosinophilic CRSwNP subtypes, lesion DCs display different T helper polarization capacities and metabolic features with potential value for stratification and prediction [111].

Therapeutically, remodeling DC metabolism reduces generation of sensitizing T cells and mitigates airway inflammation; activating AMPK, mobilizing FAO/OXPHOS, or moderately inhibiting mTOR induces RALDH^+^ and PD-L1^+^ tolDCs and up-regulates IL-10/TGF-β, thereby suppressing Th2/ILC2-driven mucosal inflammation [112].

#### Mφs: Mitochondrial homeostasis in chronic inflammation

Mφs are key regulators in AR and related airway diseases, with metabolic states directly determining direction and magnitude of inflammation [113]. In the nasal mucosal microenvironment, metabolomic and single-cell evidence shows abnormal accumulation of stress granules (SGs) in AR Mφs, accompanied by impaired efferocytosis/clearance and reduced IL-10, thereby amplifying local inflammation; in mouse and human samples, inhibiting SGs or intervening in the G3BP1 posttranscriptional axis partially restores phagocytosis and alleviates inflammation [114].

Regarding lipid metabolism, population metabolomics suggest dysregulated fatty acid pathways and altered acyl-carnitine spectra in AR and allergic airway disease; together with pediatric FA data within the allergic spectrum, downward shifts in long-chain acyl-carnitines and constrained β-oxidation align with reprogramming of Mφs toward glycolysis-dependent proinflammatory states [115]. Further studies indicate that FAO and mitochondrial homeostasis are central hubs for Mφ M1/M2 polarization and inflammatory thresholds [114]. In the amino acid dimension, changes in Arg and Gln pathways can affect the mTOR–STAT6–ARG1 axis, thereby influencing Mφ polarization and tissue repair; multi-omics and mendelian randomization (MR) studies provide additional evidence linking “metabolism and allergy” [116,117]. Translationally, restoring FAO and enhancing IL-10-related metabolic axes may offer early intervention: In pulmonary Mφs and acute lung injury models, l-carnitine improves mitochondrial function and FAO and reduces inflammatory burden, supporting its biological feasibility as a metabolic adjunct [118].

### Matrix/barrier cells

#### ECs and FBs: The glycolysis–lactate loop in tissue remodeling

During remodeling and chronic progression in type 2 inflammatory diseases, ECs and interstitial FBs undergo tightly coupled metabolic reprogramming. In airway and esophageal epithelium under allergic inflammation and hypoxia, glycolytic shift and HIF-1α-dependent rewiring occur, with up-regulation of HK2 and PKM2 and increased lactate generation, leading to differentiation blockade and barrier disruption, and promoting release of epithelial alarmins and metabolic mediators that amplify type II inflammation and transmit metabolic signals to adjacent stroma [37]. Single-cell and spatial multi-omics in human CRSwNP identify metabolically characterized EC and SC subsets [119]. On the one hand, glycolytic programs and epithelial–mesenchymal transition (EMT)-related pathways driven by IL-1β and environmental stimuli are enriched at the epithelium–stroma interface; on the other hand, specific SCs (FBs and myo-FBs) spatially colocalize with type II immune pathways, indicating tissue-level integration along a “metabolism–inflammation–remodeling” axis [120].

FBs, under IL-1β, IL-11, and TGF-β, undergo activation characterized by enhanced glycolysis with adaptive mitochondrial OXPHOS, acquiring α-smooth muscle actin (α-SMA) collagen type I α 1 chain (COL1A1) and COL3A1 fibrotic markers. PFKFB3 serves as a rate-limiting node: By elevating fructose-2,6-bisphosphate, it drives glycolytic flux, collagen synthesis, and extracellular matrix (ECM) remodeling; blocking PFKFB3 or related glycolytic steps significantly reduces fibrosis in multiple models [121]. Beyond internal mucosal barriers, the integration of metabolic and signaling networks is prominently observed in the skin, particularly in emerging conditions such as prurigo nodularis (PN). In PN lesions, activated skin FBs establish a “metabolic-neural” circuit with sensory nerve endings to sustain chronic pruritus [122]. These FBs undergo glycolytic reprogramming and release metabolic by-products that directly lower the activation threshold of neighboring sensory neurons. This neuro-metabolic crosstalk, coupled with FB-derived ECM remodeling, constitutes a self-sustaining loop that drives the characteristic hyperkeratotic nodules and persistent itch-scratch cycles in PN.

Metabolic “crosstalk” contributes to refractory phenotypes: Epithelial-derived lactate acts via GPR81 and MCT1/MCT4 on FBs, inducing α-SMA and COL1A1 and maintaining pro-fibrotic/pro-inflammatory states, establishing a positive feedback loop of “epithelial glycolysis–lactate accumulation–FB activation” [123]. From an interventional perspective, jointly targeting EC–FB metabolic pathways lowers inflammation and remodeling in allergic airway and fibrosis-related models; concurrently, inhibiting the HIF-1α–glycolysis axis down-regulates innate immune cell metabolism and functions related to type II responses, offering a testable path for integrated “metabolism–immunity–stroma” interventions [124] (Table 2).

**Table 2.** Cellular heterogeneity and immunometabolic reprogramming in type 2 inflammatory diseases

Cell type/subset	Core pathogenic/regulatory function	Major metabolic program/node	Key signaling/receptors	Representative diseases
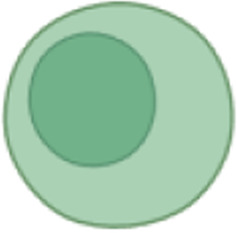 T cells	Tissue-resident and rapidly reactivating in asthma and AD; promote IgE class switching in FA; induce tolerant Treg/Tr1 during AIT/OIT	Resting TRM rely on FAO–OXPHOS; activation phase shows ↑ glycolysis or mixed metabolism; LAT1–mTORC1, PFKFB3–HIF-1α, PDK1; tolerance accompanied by ↑ NAD^+^/sirtuin, mitochondrial biogenesis, Trp–AhR	IL-33 and TSLP triggering; c-Myc-dependent nucleotide synthesis	Asthma, AD, FA, AA, EoE
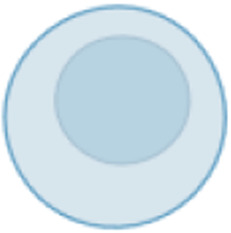 B cells/PCs	Memory reservoir rapidly class-switching to pathogenic IgE; *Tfh13* drives high-affinity IgE; tissue LLPCs maintain sensitization memory	PCs: XBP1-mediated ER stress; mTORC1-driven biosynthetic flux; tissue PCs show parallel up-regulation of glycolysis and OXPHOS	BAFF/APRIL–BCMA axis; IL-6, CXCL12 sustain niche; *Tfh13* (high IL-13/low IL-21)	FA, CRSwNP, anaphylaxis
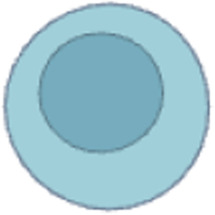 ILC2s	Rapid activation by epithelial alarmins, early amplifier of type II inflammation; key effector in steroid-resistant/severe eosinophilic asthma (SEA)	IL-33 → ↑ glucose uptake and glycolysis, ↑ ROS, mTOR-dependent; PPARγ couples FAO/OXPHOS with effector transcription	ST2 (IL-33R), IL-25R, TSLPR; NMUR1–NMU neuro-immune axis; SCFAs–GPR43; lactate–GPR81/PPARγ	SEA, eosinophilic pneumonia
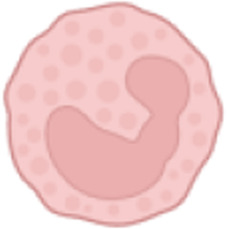 Eos	Drive airway/nasal remodeling, pruritus and barrier damage; positive feedback with epithelial IL-13 in EoE/EGE	Switch between glycolysis and OXPHOS; combined FAO sustains survival and effector output; mixed metabolic profile associates with pathogenicity	Respond to IL-5/IL-5R; act synergistically with TGF-β and IL-33 axes	Asthma, EoE, EGE, EGPA
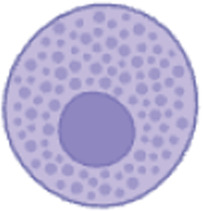 MCs	IgE–FcεRI-dependent and non-IgE (MRGPRX2)-mediated degranulation, pruritus, remodeling; contribute to vascular remodeling in childhood asthma	Environmental cues induce lipid/amino acid metabolic reprogramming; metabolism shapes receptor microdomains and sensitivity	FcεRI; MRGPRX2/B2; ORAI1/2 CRAC; PAR, C5aR, TLR2/4; PI3K/PLCγ/MAPK/NF-κB	AD, CSU, anaphylaxis, childhood asthma
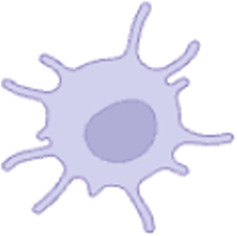 DCs	Amplify type II responses during sensitization; tolerogenic subtype suppresses aberrant Th2 via PD-L1, IL-10/TGF-β	Sensitizing: ↑ glycolysis, GLUT↑, PI3K–AKT–mTORC1, HIF-1α; tolerogenic: FAO/OXPHOS-dominant with AMPK/PPAR/NCoR1	CCR7-dependent migration; driven by epithelial alarmins IL-33/TSLP	AR, Asthma, CRSwNP
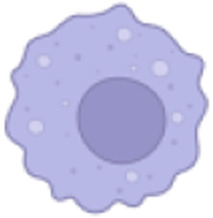 Mφs	Regulate inflammatory intensity in AR; SG aggregation suppresses efferocytosis and IL-10 production	FAO/mitochondrial homeostasis governs polarization; long-chain acyl-carnitine ↓ and β-oxidation restriction in allergic spectrum; glycolytic shift	mTOR–STAT6–ARG1 axis influenced by amino acid metabolism	AR, asthma, CRSwNP
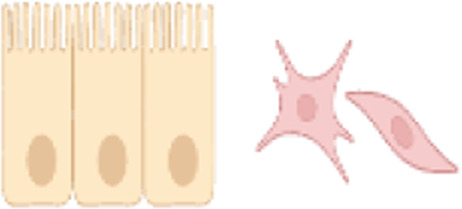 ECs and FBs	Glycolytic shift → ↑ lactate → barrier disruption and alarmin release; FB activation → ECM remodeling/fibrosis	HIF-1α-dependent rewiring; ↑ HK2/PKM2; lactate–GPR81/HCAR1 and MCT1/4 mediate crosstalk	IL-1β/IL-11/TGF-β drive fibrotic programs; IL-33/TSLP released from epithelium	CRSwNP, prurigo nodularis (PN)

## Tissue-Specific Metabolic Microenvironments

**Fig. 3.**
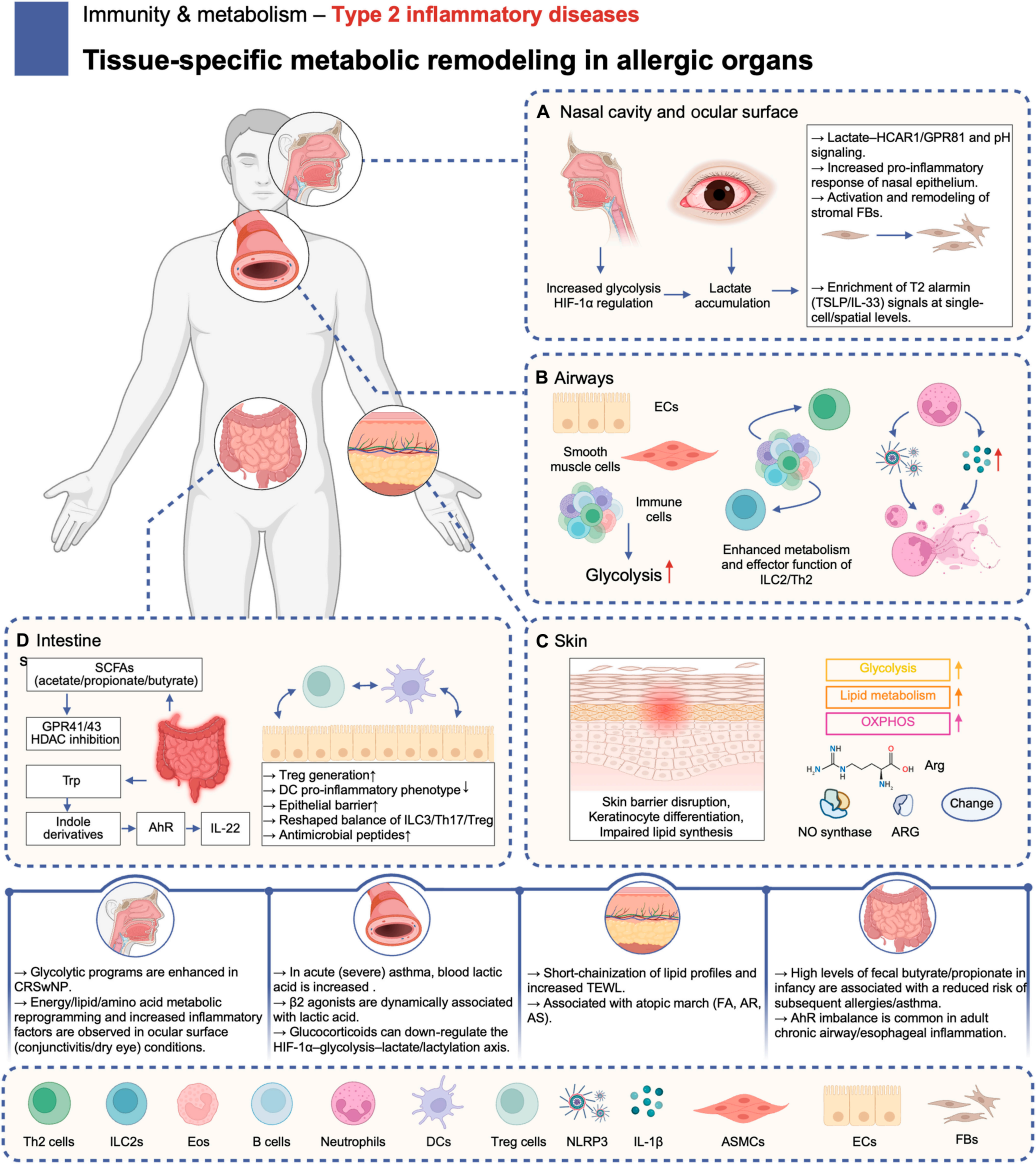
Tissue-specific metabolic remodeling in allergic organs. (A) Nasal/ocular mucosa: HIF-1α-controlled epithelial glycolysis raises lactate and activates HCAR1/GPR81–pH signaling, amplifying inflammation and fibroblast remodeling; single-cell/spatial data show enrichment of type 2 alarmins thymic stromal lymphopoietin (TSLP) and interleukin-33 (IL-33). (B) Airways: Up-regulated epithelial and smooth muscle glycolysis augments ILC2/Th2 metabolism, sustaining chronic type 2 inflammation; in severe asthma, lactate correlates with β2-adrenergic agonist exposure, while corticosteroids suppress the HIF-1α–glycolysis–lactylation axis. (C) Skin: Barrier dysfunction, aberrant keratinocyte differentiation, and lipid dysregulation co-occur with imbalanced glycolysis, FA metabolism, and OXPHOS; nitric oxide synthase (NOS) and arginine (Arg) pathways modulate immune homeostasis. (D) Gut: Short-chain fatty acids (SCFAs)—acetate, propionate, and butyrate—regulate immunometabolism via G-protein-coupled receptors 41/43 (GPR41/43) and histone deacetylase (HDAC) inhibition, promoting regulatory T (Treg) differentiation and interleukin-22 (IL-22); indoles act via AhR. Higher infant fecal SCFAs associate with reduced later allergy risk; adult eosinophilic esophagitis (EoE) shows impaired AhR signaling.

In type 2 inflammatory diseases, barrier sites—including the nasal cavity, ocular surface, airways, skin, and gut—commonly exhibit a metabolic landscape of “hypoxia–HIF-1α stabilization–enhanced glycolysis–lactate accumulation”. On the one hand, this axis supplies rapid energy to damaged or stressed cells; on the other hand, lactate and epigenetic layers such as histone lactylation act as a “metabolic language” that reshapes effector thresholds of immune cells and SCs, thereby amplifying inflammation and tissue remodeling [125]. In the airways, HIF-1α-driven glycolysis synergizes with epithelial alarmin networks, augmenting the pro-inflammatory output of Th2 and ILC2s while, via the FB PFKFB3 node and the lactate–GPR81 circuit, driving collagen synthesis and ECM remodeling—constituting a metabolic fulcrum for structural remodeling [126]. In the skin, abnormalities in stratum corneum lipids such as CER and FFAs, together with defects in epithelial differentiation, disrupt the barrier and promote a “transepidermal sensitization–chronic inflammation” cascade. Type II cytokines down-regulate synthesis/elongation of very-long-chain fatty acids, supporting a causal coupling of “lipid profile–barrier function–inflammation” [127]. In the gut, microbial metabolites connect diet with immune tolerance: SCFAs promote Tregs, stabilize the epithelium, and suppress sensitization via GPCRs and histone deacetylase (HDAC) inhibition; the *Trp–indole–AhR–IL-22* axis maintains mucosal homeostasis and attenuates inflammation. In early life, adequacy of SCFAs and indoles associates with reduced subsequent risk of allergy or asthma [128]. In CRSwNP, single-cell and spatial analyses reveal broadly enhanced glycolytic programs with enrichment of type II inflammatory signals in ECs and immune cells; together with FB PFKFB3 and lactate–GPR81 evidence, these findings place a cross-cell “glycolysis–lactate–remodeling” circuit at the core of persistent nasal inflammation and structural change. Metabolomic and proteomic studies on the ocular surface likewise suggest reproducible changes in energy, lipid, and amino acid pathways, although cellular and spatial resolution still needs improvement (Fig. 3).

### Nasal cavity and ocular surface

As barrier tissues first exposed to external allergens and environmental stimuli, the nasal cavity and ocular surface depend on precise interplay among ECs, the local microenvironment, and metabolic networks for immune homeostasis. In AR, eosinophilic CRSwNP, and AC, metabolic remodeling with “enhanced glycolysis–lactate accumulation” is a key driver of chronic inflammation and immune imbalance. Elevated glucose in nasal secretions of CRSwNP can push NECs toward glycolysis and amplify pro-inflammatory responses; nasal FBs and remodeling-related pathways likewise show up-regulation of glycolytic nodes such as HK2 and PFKFB3 under HIF-1α control [129]. In CRSwNP, ECs, SCs, and immune cells broadly exhibit enhanced glycolytic programs, increased intercellular interactions, and enrichment of alarmin signals related to type II inflammation [130]. Lactate also acts as an active signal in the nasal cavity and on the ocular surface, reshaping immunity via GPR81/HCAR1 and pH-dependent routes: It may suppress or retune activation, chemotaxis, and cytokine release in effector cells such as MCs, and may shift thresholds of local amplification circuits [131,132]. By contrast, mechanistic evidence for the ocular surface remains at an early stage. Current metabolomic/proteomic data show reproducible alterations in energy, lipid, and amino acid metabolism in tear fluid and the corneo-conjunctiva during AC and dry eye-related inflammation, correlating with increased IL-6, IL-8, and TNF-α. However, most studies analyze bulk metabolomes with limited spatial/cellular resolution, constraining causal inference and precise target localization [133].

Overall, key gaps remain in the “metabolism–immunity” interplay at the nasal cavity and ocular surface—specifically, how metabolites such as lactate, SCFAs, and Gln shape functional fates across MCs, Eos, neutrophils, DCs, and NECs, and how these pathways bidirectionally couple with epithelial alarmins and receptor signaling. Based on existing evidence, metabolism-focused interventions already show potential in models of nasal mucosal remodeling and immune imbalance; for ocular surface disease, longitudinal cohorts and spatially resolved multi-omics will be critical to validate pathogenicity along the metabolic–immune axis and to guide metabolic-targeted therapies [134].

### Airways

Local hypoxia in the airways stabilizes and activates HIF-1α, guiding bronchial ECs, airway smooth muscle cells (ASMCs), and multiple immune cell types into glycolysis-centered metabolic reprogramming with marked local lactate generation. This “HIF-1α–glycolysis–lactate” axis not only provides rapid energy for injury or stress but also serves as a “metabolic language” within signaling networks that re-sculpts effector cell fate and function, amplifying or sustaining inflammation and remodeling [7,135]. In canonical type II phenotypes, HIF-1α-driven glycolysis enhances the metabolic activity and pro-inflammatory output of ILC2s and Th2 and synergizes with epithelial-barrier/differentiation defects. In neutrophil-dominant phenotypes or those with intermittent hypoxia, HIF-1α can also activate the NOD-like receptor family pyrin domain-containing protein 3 (NLRP3)–IL-1β-dependent pyroptotic pathway, promoting neutrophil recruitment and persistent inflammation and thereby aggravating smooth muscle hyperplasia, inflammatory infiltration, and structural remodeling [136]. Lactate is not an inert “metabolic waste”, but a key immunoregulator: (a) Via epithelial-derived signals, it can act on alveolar and airway Mφs to retune inflammatory intensity, and (b) at the epigenetic level, “protein lactylation” rewires transcriptional and metabolic circuits in cells such as Mφs, shaping the airway immune microenvironment at the molecular scale [137,138]. Clinically, elevated blood lactate observed in acute or severe asthma highlights the real-world relevance of metabolic dysregulation during exacerbations and suggests complex relationships between β_2_ adrenergic receptor agonists (β₂-agonists) and lactate dynamics [139]. In addition, ICS not only suppress inflammation but also down-regulate the “HIF-1α–glycolysis–lactate/lactylation” axis at multiple levels—for example, by inhibiting HIF-1α-dependent glucose uptake and glycolysis in Mφs—providing a metabolic explanation for part of their efficacy [140]. Targeting HIF-1α, glycolytic checkpoints, or lactate/lactylation signaling may complement therapy for refractory, severe, or phenotype-specific asthma; however, the spatiotemporal distribution of airway lactate, intercellular metabolic crosstalk among ECs, SMCs, Mφs, ILC2s, Eos, and neutrophils, and their contributions across clinical subtypes such as AERD and OA require finer stratification and target validation via cohorts combined with single-cell and spatial omics.

### Skin

Integrity of the cutaneous barrier is jointly maintained by the stratum corneum lipidome—particularly CER and FFAs—and keratinocyte (KC) differentiation programs. When differentiation [e.g., filaggrin (FLG)] or lipid synthesis/modification pathways are impaired, measurable alterations in lipid composition and lamellar structure disrupt the “brick-and-mortar” barrier, leading to transepidermal water loss and increased permeability, which in turn trigger and maintain inflammation—laying the molecular groundwork for AD onset and chronicity [141,142]. In AD lesions, lipidomics shows decreased total CER, a shift toward shorter chain species, and reduced average FFA chain length, paralleling barrier dysfunction. Mechanistically, IL-4/IL-13 down-regulate key elongases such as elongation of very long-chain fatty acids protein 3 (ELOVL3) and ELOVL6 via STAT6, directly driving “short-chain” lipid profiles and barrier breakdown and clarifying the mutual amplification of immune and lipid imbalance [143]. Beyond genetics and immunity, epigenetic and posttranscriptional control within KCs also links “metabolism–barrier–inflammation”. For example, N^6^-methyladenosine (m^6^A) modification stabilizes ELOVL6 mRNA through the methyltransferase-like 3 (METTL3)–insulin-like growth factor 2 mRNA-binding protein 3 (IGF2BP3) axis, maintaining fatty acid elongation and lipid homeostasis; when impaired, lipid metabolism is re-routed and inflammatory/chemotactic signals are amplified [144]. These epithelial-intrinsic metabolic changes determine local barrier integrity and, via “transepidermal sensitization”, promote IgE responses to food antigens, closely related to the “atopic march” from AD to FA, rhinitis, and asthma [145]. In parallel, allergic contact dermatitis (ACD) exhibits partially overlapping yet distinct metabolic reprogramming: Contact sensitizers induce simultaneous up-regulation of glycolysis, fatty acid metabolism, mitochondrial OXPHOS, and altered Arg metabolism in KCs—pathways that not only supply precursors and energy for inflammation but also shape antigen presentation, barrier repair, and clinical outcomes. Targeting specific lipid-metabolic enzymes (e.g., ELOVL) or lactate transport/receptor nodes may complement current immunomodulatory and barrier-repair therapies [146].

### Gut

The gut microbiota ferments dietary fiber into SCFAs—primarily acetate, propionate, and butyrate—and converts Trp into multiple indole derivatives. These metabolite classes form key axes linking the intestinal ecosystem with systemic allergic IgE and type II inflammatory phenotypes. SCFAs, by activating GPCRs (GPR41/FFAR3, GPR43/FFAR2) and inhibiting HDACs, promote peripheral Tregs, suppress pro-inflammatory DC phenotypes, and strengthen the epithelial barrier, thereby lowering early sensitization to dietary antigens and mitigating IgE-dependent allergic responses; evidence includes SCFA-driven peripheral colonic Tregs and cohort data associating higher infant fecal butyrate/propionate with reduced later allergy/asthma risk [147]. Microbiota-derived Trp metabolites such as indole-3-aldehyde (I3A) and indole-3-carboxaldehyde (I3CA) act as endogenous AhR ligands to up-regulate epithelial IL-22, rebalance group 3 innate lymphoid cells (ILC3s)/Th17/Tregs, and maintain the mucosal barrier and antimicrobial peptides; exogenous IL-22 or enhanced indole metabolism boosts intestinal AhR activity, improves the barrier, and attenuates inflammation [148,149]. Notably, SCFAs and indoles do not act in isolation: Together, they modulate DC antigen presentation and metabolic programs, epithelial permeability, and antimicrobial peptide profiles, thereby influencing distal allergic phenotypes via the “gut–lung/skin” axes; imbalance of the Trp–AhR pathway is more frequent in adult chronic airway or esophageal eosinophilic inflammation [150]. Interventions that increase dietary fiber and butyrate/propionate-producing taxa, deliver site-directed butyrate prodrugs or particles, or supplement specific indole molecules can improve the barrier and immune tolerance and reduce allergy severity; however, efficacy across allergic subtypes/age groups, dose–response relationships, and long-term safety require validation in larger longitudinal cohorts and randomized controlled trials (RCTs) [151] (Table 3).

**Table 3.** The tissue-specific immunometabolic landscape: Shaping local microenvironments and remodeling trajectories

Site/tissue	Core metabolic axis/node	Main cellular/pathway effects	Metabolite–signal coupling	Clinical/phenotype links
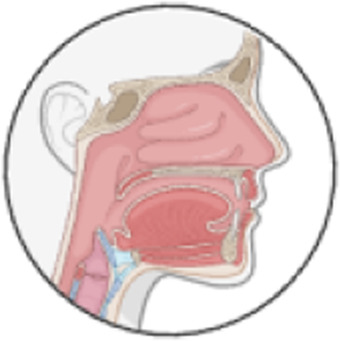 Nasal cavity/ocular surface	Glycolysis↑ (HK2, PFKFB3) via HIF-1α → lactate build-up; lactate–HCAR1/GPR81 and pH signaling	Nasal epithelial pro-inflammation↑; stromal fibroblast activation and remodeling; single-cell/spatial data reveal T2 alarmin enrichment (TSLP/IL-33)	Lactate modulates MC and other effector activation/chemotaxis/cytokines; sets thresholds of local amplification loops	CRSwNP/eCRSwNP show widespread glycolytic programs; ocular surface (conjunctivitis/dry eye) exhibits energy/lipid/amino acid rewiring with cytokine elevation
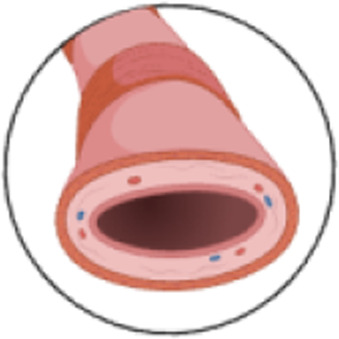 Airway	Hypoxia→ HIF-1α↑ → glycolysis↑ → lactate accumulation; protein lactylation	Glycolysis up in epithelium/smooth muscle/immune cells; in T2 endotype: ILC2/Th2 metabolism and output↑; in neutrophilic endotypes: NLRP3–IL-1β pyroptosis	Lactate as immunomodulator (acts on Mφs; epigenetic lactylation reprograms transcription)	Acute/severe asthma shows elevated blood lactate; β2-agonist interactions; ICS down-tunes HIF-1α–glycolysis–lactate/lactylation axis
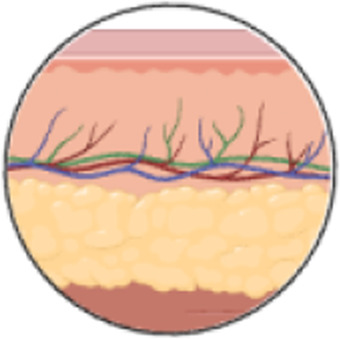 Skin	Stratum corneum lipid imbalance (CER↓, shorter FFAs); STAT6→ ELOVL3/6↓; METTL3–IGF2BP3 stabilizes ELOVL6 (m6A)	KC differentiation/lipid synthesis defects → barrier failure and epicutaneous sensitization; in ACD, glycolysis/FA metabolism/OXPHOS co-up-regulated; arginine metabolism altered	Type 2 cytokines (IL-4/IL-13) drive “short-chain” lipidome; metabolic reprogramming sets antigen presentation, repair, and thresholds	Shorter lipid chains correlate with ↑TEWL; linked to atopic march (FA, rhinitis, asthma)
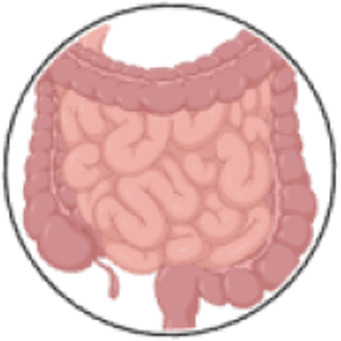 Gut	SCFAs (acetate/propionate/butyrate) → GPR41/43 and HDAC inhibition; Trp→ indoles→ AhR→ IL-22	↑Tregs, ↓DC pro-inflammatory profile, ↑epithelial barrier; rebalance ILC3/Th17/Treg	SCFAs and indoles co-tune DC metabolism/presentation and epithelial permeability → “gut–lung/skin” axis	Higher infant butyrate/propionate associates with lower later allergy/asthma; adult chronic airway/EoE often show AhR imbalance

## A Cross-Disease Metabolic–Signaling Atlas

Cross-organ and cross-phenotype evidence indicates that AS, AR, AD, FA, CRSwNP, and AC can generally be subsumed under an actionable axis of “hypoxia–HIF-1α–glycolysis–lactate–immunometabolic remodeling”. Local hypoxia stabilizes site-specific HIF-1α and drives glycolysis and lactate generation in ECs, SMCs, SCs, and immune cells; lactate, via receptors/transporters such as GPR81/HCAR1 and MCT1/MCT4 and through histone lactylation at the epigenetic level, “translates” metabolic state into signals of inflammatory amplification or persistence [7]. In AS, this axis synergizes with epithelial alarmin networks to synchronously boost the energy supply and effector output of ILC2s and Th2 cells, while FBs acquire pro-fibrotic and ECM-remodeling phenotypes through the PFKFB3 node and a “lactate–GPR81–HIF-1α” feedforward loop, linking structural change with refractory subtypes [152].

**Fig. 4.**
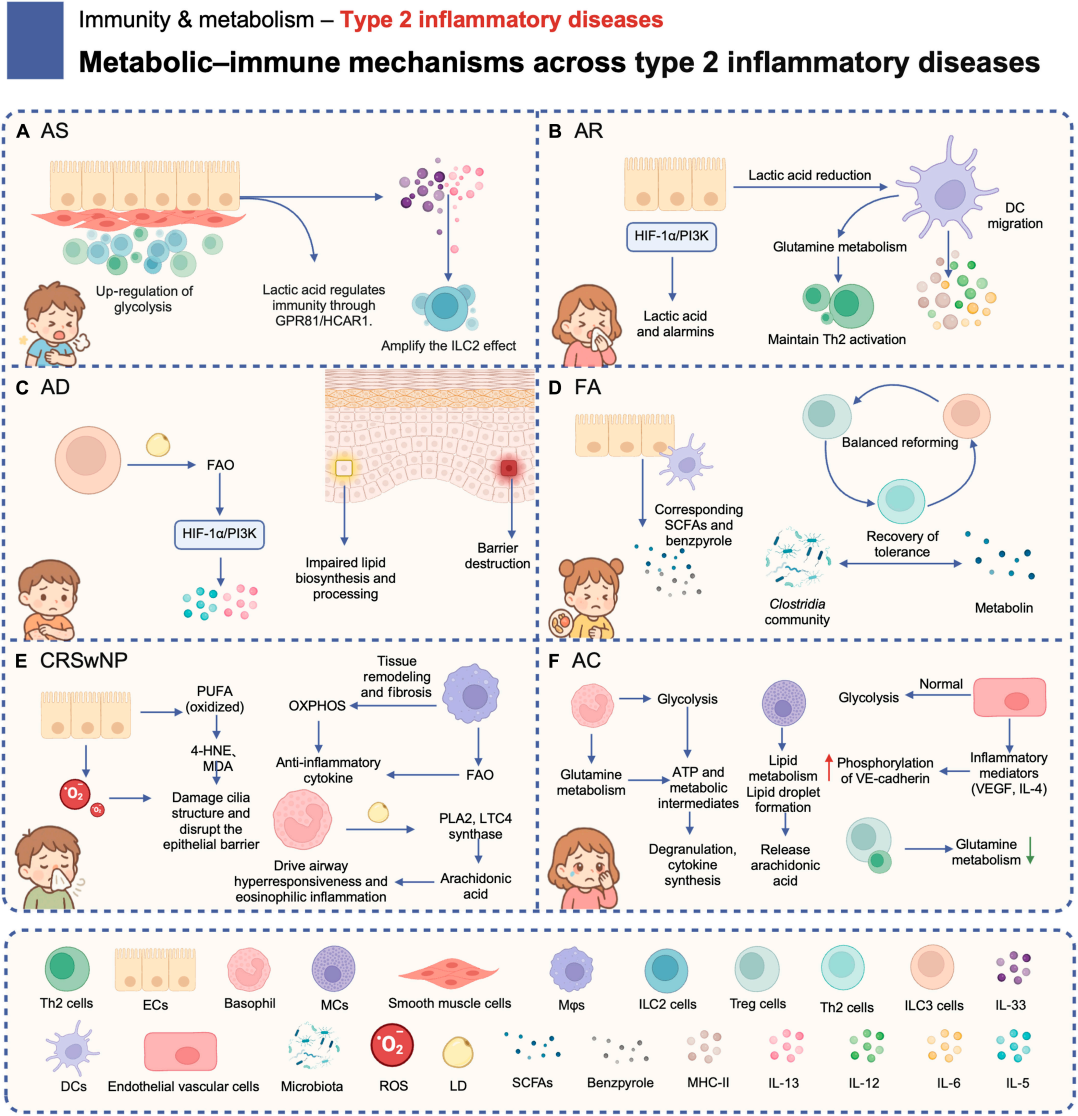
Metabolic–immune mechanisms across type 2 inflammatory diseases. (A) Allergic asthma (AS): Glycolysis-derived lactate activates ILC2s via GPR81/HCAR1, amplifying type 2 inflammation. (B) Allergic rhinitis (AR): HIF-1α/PI3K signaling drives lactate and alarmin production, sustaining Th2 activation; glutamine-metabolism imbalance perturbs local homeostasis. (C) Atopic dermatitis (AD): Abnormal FAO and HIF-1α/PI3K signaling impair lipid biosynthesis and barrier integrity. (D) Food allergy (FA): Clostridia-derived short-chain fatty acids (SCFAs) and benzopyrroles foster tolerance; restoring microbial metabolites re-establishes immune balance. (E) Chronic rhinosinusitis with nasal polyps (CRSwNP): ROS and oxidized polyunsaturated fatty acid (PUFA) products—e.g., 4-hydroxynonenal (4-HNE) and mycophenolic acid (MPA)—damage cilia and barrier; FAO/OXPHOS dysregulation contributes to remodeling, and phospholipase A_2_ (PLA_2_) and leukotriene C_4_ (LTC_4_) pathways promote fibrosis. (F) Allergic conjunctivitis (AC): Heightened glycolysis and glutamine metabolism drive ATP generation, lipid-droplet formation, and vascular endothelial growth factor (VEGF)/interleukin-4 (IL-4) release, leading to degranulation and barrier disruption.

In AR, a glycolytic shift and lactate efflux in nasal epithelium not only elevate the local inflammatory threshold but also tune the presentation/polarization potential of DCs via lactate–GPR81. Meanwhile, DC migration and antigen processing still rely on mitochondrial flux supported by the “Gln–α-ketoglutarate (α-KG)” branch, indicating that the “glycolysis–lactate–GPR81” and “Gln–TCA/OXPHOS” parallel systems jointly shape the immunologic backdrop of AR. In the skin, IL-4/IL-13 down-regulate ultra-long-chain fatty acid elongation and SL remodeling pathways (e.g., ELOVL3 and ELOVL6), leading to “short-chain” shifts in stratum corneum lipid profiles and barrier disruption, forming a pathogenic coupling of “lipid metabolism–barrier–immunity” [142]. In FA, the gut microbiota connects diet with immune tolerance: SCFAs promote Tregs and barrier homeostasis via GPCRs and HDAC inhibition, while the “Trp–indole–AhR–IL-22” axis maintains mucosal stability and lowers sensitization risk; infant studies suggest that elevating propionate/butyrate and indole-type ligands is a plastic intervention route [153]. In CRSwNP/CRS, single-cell and spatial transcriptomics reveal broadly enhanced glycolytic programs and type II inflammatory signals across ECs, SCs, and immune cells, together with LPO, LD formation, and FAO programs, constituting a metabolic scaffold of “smoldering inflammation–remodeling” [154] (Fig. 4).

### AS

In AS, recurrent inflammation and narrowing in airway mucosa produce local hypoxia that stabilizes and activates HIF-1α, transcriptionally up-regulating key glycolytic enzymes (HK2, PKM2, and LDHA) in ECs, ASMCs, and type II immune cells—especially ILC2s and Th2—establishing a Warburg-like metabolism that persists even under oxygen availability and rapidly accumulates lactate [135]. Upstream signals such as IL-33/IL-13 further amplify this circuit, synchronously enhancing bioenergetic supply and effector functions (IL-5 and IL-13 production) in ILC2s. Translational evidence indicates that pharmacologic inhibition of glycolysis (e.g., 2-deoxy-glucose), blockade of HIF-1α, or iron restriction that suppresses mitochondrial oxidation and stabilizes HIF-1α can all reduce ILC2 metabolic activity and proinflammatory output, alleviating airway hyperreactivity and inflammation [155]. In parallel, lactate rises from “metabolic waste” to immune signal: Via GPR81/HCAR1 and pH-dependent mechanisms, it modulates chemotaxis, activation, and cytokine profiles of innate and adaptive immune cells; as a donor for “protein lactylation”, it remodels chromatin and transcriptional networks in cells such as Mφs, thereby reprogramming the airway immune microenvironment at the molecular scale [131].

Overall, asthma features an actionable pathway of “hypoxia–HIF-1α–glycolysis–lactate–immunometabolic remodeling”, with one arm feeding ILC2- and Th2-driven type II inflammation and airway remodeling, and the other reinforcing the inflammatory niche via lactate–receptor/lactylation signaling. Targeting this axis—through HIF-1α inhibition, glycolytic rate-limiting enzyme blockade, or modulation of lactate/GPR81–lactylation—may serve as a complementary strategy for refractory or phenotype-specific asthma [156].

### AR

During the onset and recurrence of AR, NECs, DCs, and memory Th2 cells form a bidirectionally coupled network centered on glucose and Gln metabolism and inflammatory signaling.

First, allergens trigger metabolism–inflammation coupling on susceptible or slightly damaged epithelial barriers [157]. NECs display a glycolytic shift and enhanced glucose uptake; under hypoxia–HIF-1α and PI3K amplification, they release lactate and epithelial alarmins, heightening innate activation and proinflammatory responses [158]. Lactate and the acidified microenvironment are not inert backgrounds but act via GPR81/HCAR1 or MCTs on neighboring immune cells. In related studies, lactate–GPR81 signaling down-regulates major histocompatibility complex class II (MHC-II) and IL-12/IL-6 in DCs, alters their migration and costimulatory profiles, and thereby influences their capacity to initiate and consolidate Th2 responses [159].

Second, in the “antigen presentation–T cell polarization” step, DCs—despite a highly glycolytic milieu—still rely on “MHC-II”-centered maintenance of mitochondrial energy, reducing equivalents and biosynthetic substrates to support antigen processing/presentation and CCR7-dependent migration; when Gln uptake or utilization is impaired, DC presentation and Th2-promoting capacity drop markedly [160].

In summary, the “glycolysis–lactate–GPR81” axis and the “Gln–TCA/OXPHOS” axis run in parallel within the nasal epithelium–DC–Th2 circuit of AR: The former sets the local inflammatory threshold and DC activation “background”, whereas the latter provides metabolic support for DC presentation and Th2 polarization [161]. Accordingly, targeting HIF-1α/glycolysis, lactate–GPR81 signaling, or Gln-metabolic nodes in DCs may offer stratified interventions complementary to existing immunotherapies [162].

### AD

In AD, the triad “lipid metabolism–barrier–immunity” has emerged as a pathogenic hub. At steady state, KCs maintain the “brick-and-mortar” structure and low-permeability barrier of the stratum corneum through synthesis and remodeling of long/very-long-chain fatty acids and SLs. In AD cohorts and models, IL-4/IL-13 within type II inflammation reduce key elongases and related pathways downstream of STAT6 (e.g., ELOVL3 and ELOVL6), shortening fatty acid chain length, disturbing CER composition, and altering FFA profiles, thereby causing transepidermal water loss, barrier disruption, and amplified immune activation [142,143]. Such lipidomic abnormalities are observed in FLG-deficient mice and human lesions and align with impaired barrier function [163]. Beyond synthesis, diseased epidermis shows imbalance in oxidation and catabolism: Heightened mitochondrial and global oxidative stress and accumulation of LPO products associate with inflammatory amplification; in some models, peroxisomal β-oxidation increases together with glycolysis, further fueling lipid disequilibrium and a self-sustaining inflammatory loop [164]. m6A modification, via the METTL3–IGF2BP axis, stabilizes lipid-metabolism mRNAs and mediates an “m6A–lipid metabolism–inflammation” pathway; when impaired, epithelial lipid homeostasis collapses, amplifying inflammatory and chemotactic signals and explaining certain “refractory barrier defects” at the molecular level [144]. On the immune side, AD-related innate/adaptive type II immunity is lipid-dependent: ILC2s require fatty acid uptake, LD formation, and mitochondrial FAO in sensitization and tissue repair/remodeling contexts; their LD–DGAT1/PPAR axis and IL-33-induced assimilation/oxidation programs correlate with pathogenic expansion, and quantitative frameworks for fatty acid uptake/storage/oxidation in primary ILC2s have been established [165]. Altogether, stratified co-targeting of “epithelial lipid synthesis/processing (ELOVL/SLs)–peroxisome/oxidative stress” and “ILC2 lipid metabolism (LD/FAO/PPAR)” may complement barrier-repair and biologic strategies and inform companion diagnostics [165].

### FA

FA typifies a “gut–immunity” imbalance in which the metabolic–signaling network is shaped by the microbiota and its metabolites. Commensals ferment dietary fiber into SCFAs (acetate, propionate, butyrate) and convert Trp into indole ligands [indole-3-acetic acid (IAA), indole-3-carboxaldehyde (IAld), etc.], acting on epithelium, DCs, and T cells to maintain immune tolerance and barrier homeostasis.

At steady state, SCFAs serve as energy substrates and, via GPCRs (FFAR2/GPR43, GPR109A, etc.) and HDAC inhibition, up-regulate peripheral Tregs and lower Th2 thresholds [166]. Related studies suggest that higher early-life exposure to butyrate/propionate associates with reduced later allergy risk [167]. In parallel, microbiota-derived indoles activate the AhR–IL-22 axis, enhancing epithelial antimicrobial peptides and barrier function and rebalancing ILC3/Th17/Tregs, thereby strengthening oral tolerance [149]. Dysbiosis with reduced SCFAs and disturbed indole spectra is common in allergic subjects and models; fecal microbiota from healthy infants, but not from cow’s milk-allergic infants, prevents dietary antigen allergy in mice, and Clostridia consortia or their metabolites restore tolerance pathways (Tregs, IgA, epithelial permeability) [168]. Interventions include micellar/prodrug butyrate, which restores mucosal homeostasis and reduces allergy in murine peanut models [169]; supplementation with IAA or IAld activates AhR and repairs barriers to attenuate allergic phenotypes even without full microbiota reversal [170]. In summary, the 2 axes “SCFAs–GPR/HDAC–Tregs/barrier” and “indole–AhR–IL-22/barrier”, coupled to DC presentation programs, govern maintenance and breakdown of oral tolerance; precision interventions may raise butyrate/propionate-producing taxa, deliver SCFAs locally, supplement/induce indole-type AhR ligands, and be combined with OIT or barrier-repair strategies.

### CRSwNP

The CRSwNP lesion microenvironment exhibits marked “metabolic reprogramming–immune coupling”. On the one hand, ECs, SCs, and immune cells show enhanced glycolysis and lipid-metabolism disequilibrium with spatial colocalization of type II inflammation; on the other hand, FAO, LDs, and ROS together constitute a metabolic scaffold sustaining chronic inflammation and tissue remodeling [154]. Studies indicate that epithelial LPO and up-regulation of fatty acid transport/metabolism genes (e.g., SLC27A2/FATP2 and ALOX15) correlate with disease severity and IL-4/IL-13 signaling, and that LPO directly impairs cilia/barrier function and amplifies proinflammatory networks [171]. Among immune cells, M2-like Mφs tend to rely on FAO–OXPHOS for survival and regulatory phenotypes, while LD-enriched Eos act as “depots” for energy and lipid signaling and as platforms for eicosanoid synthesis stabilized by perilipin 2/3 (PLIN2/3), linking residency, chemotaxis, and proinflammatory output. By modulating mitochondrial function, ROS–NF-κB/ALOX15 pathways, and cell-death thresholds, these nodes determine the chronic inflammatory niche across “Eos–Mφ–epithelium/stroma”. Taken together, on top of standard biologics, combined lipid-metabolism targeting—such as inhibiting FATP2/LPO, calibrated FAO, or interfering with LD biogenesis and the glycolytic axis—may mitigate mucosal remodeling and inflammatory persistence; in parallel, metabolic–inflammatory fingerprints such as LPO markers and the ALOX15 axis hold potential for stratification and companion diagnostics [172].

### AC

The research focus in AC is shifting from a classic “cytokine–receptor” paradigm to “spatial metabolism–immune interplay”. Local hypoxia and gradients of glucose, lipids, and lactate stabilize HIF-1α and up-regulate mTORC1, with PFKFB3 accelerating glycolytic rate-limiting steps; fructose-2,6-bisphosphate further elevates phosphofructokinase-1 flux. This not only boosts ATP/NADPH generation in MCs and Eos and activates the ROS–NF-κB axis and arachidonic acid mediator release but also perturbs endothelial aerobic glycolysis to disrupt VE-cadherin adhesion, increasing permeability and exudation. PFKFB3 inhibition stabilizes the barrier and reduces exudation and neovascular-related inflammation, suggesting a shared metabolic target for the ocular surface and airways [173].

Meanwhile, mitochondrial programs driven by FAO and PPAR family members (PPAR-γ/PPAR-α) can “ease the throttle” along the Th2/ILC2s axis. In IL-33/TSLP-driven type II settings, tuning fatty acid uptake and β-oxidation limits ILC2 proliferation and IL-5/IL-13 production, alleviates bronchial hyperreactivity, and promotes regulatory immunometabolic remodeling [3]. Beyond glucose and lipids, Gln metabolism is likewise critical for barrier epithelium and T cell lineage decisions: Epithelial GLS1-dependent Gln catabolism supplies nucleotides/lipids and epigenetic substrates (α-KG or acetyl-CoA) and can up-regulate alarmins IL-33 and TSLP; on the T cell side, GLS1 inhibition down-regulates HIF-1α and glycolysis and suppresses proinflammatory differentiation, supporting the feasibility of “decoupling Gln–glycolysis” [174].

Metabolic intermediates act as “second messengers” to fine-tune inflammation. Lactate, exported via MCT4 and acting on HCAR1/GPR81, resets innate/adaptive immune thresholds—overall favoring immunosuppression and Tregs stability—yet high lactate and lactylation signaling also relate to refractory inflammation and treatment resistance; thus, inhibiting MCT4 and blocking GPR81 are considered strategies to reset local immune tone [175]. In addition, the succinate–SUCNR1 pathway can amplify IgE–FcεRI and bronchoconstrictive responses across MCs, ECs, and DCs, wiring metabolic dysregulation directly to type II inflammation; by contrast, itaconate and its lipophilic derivative 4-OI act as “metabolic brakes”, alkylating sensors to activate NRF2 and suppress NF-κB and succinate dehydrogenase (SDH)-driven proinflammatory circuits, exhibiting dual anti-inflammatory and antioxidative effects in multiple models and offering translatable molecular footholds for local delivery or systemic adjuncts [176] (Table 4).

**Table 4.** Clinical immunometabolic endotypes: Mapping core metabolic axes to disease pathogenesis and phenotypes

Disease/endotype	Core metabolic axis/node	Key cells and pathways	Metabolite–signal coupling	Clinical/phenotype links
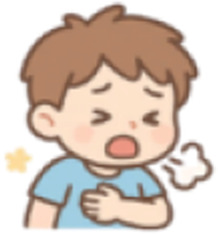 AS	Hypoxia→ HIF-1α↑ → glycolysis (HK2/PKM2/LDHA) → lactate; Warburg-like; protein lactylation	Epithelium/smooth muscle/ILC2/Th2 glycolysis↑; IL-33/IL-13 amplify ILC2s; lactate tunes Mφs and local immunity	Lactate acts via GPR81/HCAR1 and lactylation to re-program the airway niche	Exacerbation-phase lactate rises; ICS down-regulates HIF-1α–glycolysis–lactate/lactylation
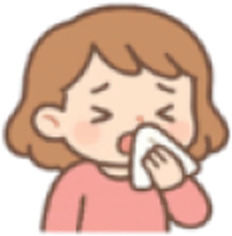 AR	Glycolytic shift→ lactate (GPR81/HCAR1, MCTs); Gln→ α-KG → TCA/OXPHOS supports DCs	Nasal epithelium HIF-1α/PI3K→ lactate + alarmins; lactate down-tunes DC MHC-II/IL-12/IL-6 and migration; DC Gln metabolism maintains Th2 priming	Lactate–GPR81 resets DC/Th2 thresholds; Gln metabolism couples presentation and polarization	Local acidification sets inflammation thresholds and relapse tendency
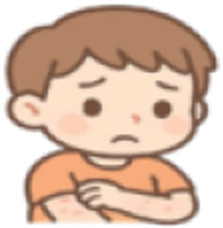 AD	STAT6→ ELOVL3/6↓→ “short-chain” lipids; m6A (METTL3–IGF2BP) stabilizes ELOVL6; peroxisomal β-oxidation/oxidative stress↑	KC lipid biosynthesis/processing defects → barrier failure; ILC2 lipid-droplet–FAO–PPAR axis supports IL-5/IL-13	Oxidized lipids as damage/inflammatory signals; lipidome shifts amplify type 2 immunity	↑TEWL with CER/FFA chain shortening; FLG defects synergize with metabolic rewiring
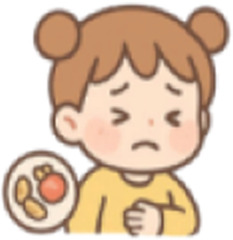 FA	SCFAs→ GPR/HDAC → ↑Tregs/lower Th2 thresholds; indoles→ AhR→ IL-22/barrier↑	Epithelial/DC/T cell responses to SCFAs and indoles; ILC3/Th17/Treg rebalance; Clostridia/metabolites restore tolerance	SCFAs and indoles shape DC metabolism and epithelial permeability to determine oral tolerance	Early butyrate/propionate associates with lower risk; allergic cohorts show ↓SCFAs/altered indoles
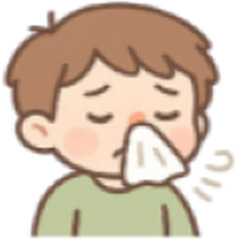 CRSwNP	Glycolysis↑ + lipid dysregulation; lipid peroxidation and FATP2/ALOX15↑; FAO/lipid droplets/ROS provide a “metabolic scaffold”	Epithelial lipid peroxidation impairs cilia/barrier; M2-like Mφs FAO–OXPHOS; eosinophil lipid droplets as eicosanoid platforms	ROS–NF-κB/ALOX15 and LD programs maintain chronic inflammation and remodeling	Spatial colocalization of metabolism–inflammation correlates with severity and IL-4/IL-13 signals
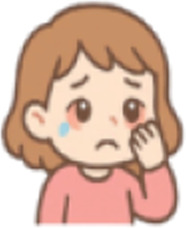 AC	HIF-1α–mTORC1→ PFKFB3↑ (F-2,6-P2↑) → glycolysis “accelerated”; FAO–PPAR “decelerates” ILC2/Th2; GLS1–Gln axis; lactate–MCT4–HCAR1; SUCNR1; itaconate/4-OI→ NRF2	MC/Eos effector programs fueled by metabolism; endothelial aerobic glycolysis perturbs VE-cadherin→ exudation; epithelial GLS1 raises IL-33/TSLP; T cell GLS1 inhibition lowers HIF-1α/glycolysis	Lactate/GPR81 resets immune tone; succinate–SUCNR1 amplifies IgE–FcεRI and bronchoconstriction; itaconate as “metabolic brake” via NRF2	PFKFB3 inhibition lowers exudation and neovascular-linked inflammation; metabolic axes link to refractoriness

## Translational Opportunities and Clinical Outlook

Current therapeutic strategies for type 2 inflammatory diseases have been revolutionized by biologics targeting key cytokines (e.g., IL-4, IL-5, and IL-13) and IgE, such as dupilumab, mepolizumab, and omalizumab. While these “top-down” approaches effectively suppress specific inflammatory axes, a significant proportion of patients remain refractory or experience relapse, often due to deep-seated metabolic reprogramming that sustains tissue-resident memory cells and structural remodeling. Emerging “bottom-up” metabolic interventions aim to complement existing biologics by resetting the underlying bioenergetic rheostats of immune cells. For instance, rather than merely neutralizing IL-5, metabolic modulators could potentially target the HIF-1α–glycolysis axis to disrupt the survival of tissue-persistent Eos. This shift from broad immunosuppression to precise metabolic “rewiring” offers a dual advantage: enhancing the efficacy of current biologics and providing a salvage pathway for steroid-resistant endotypes.

**Fig. 5.**
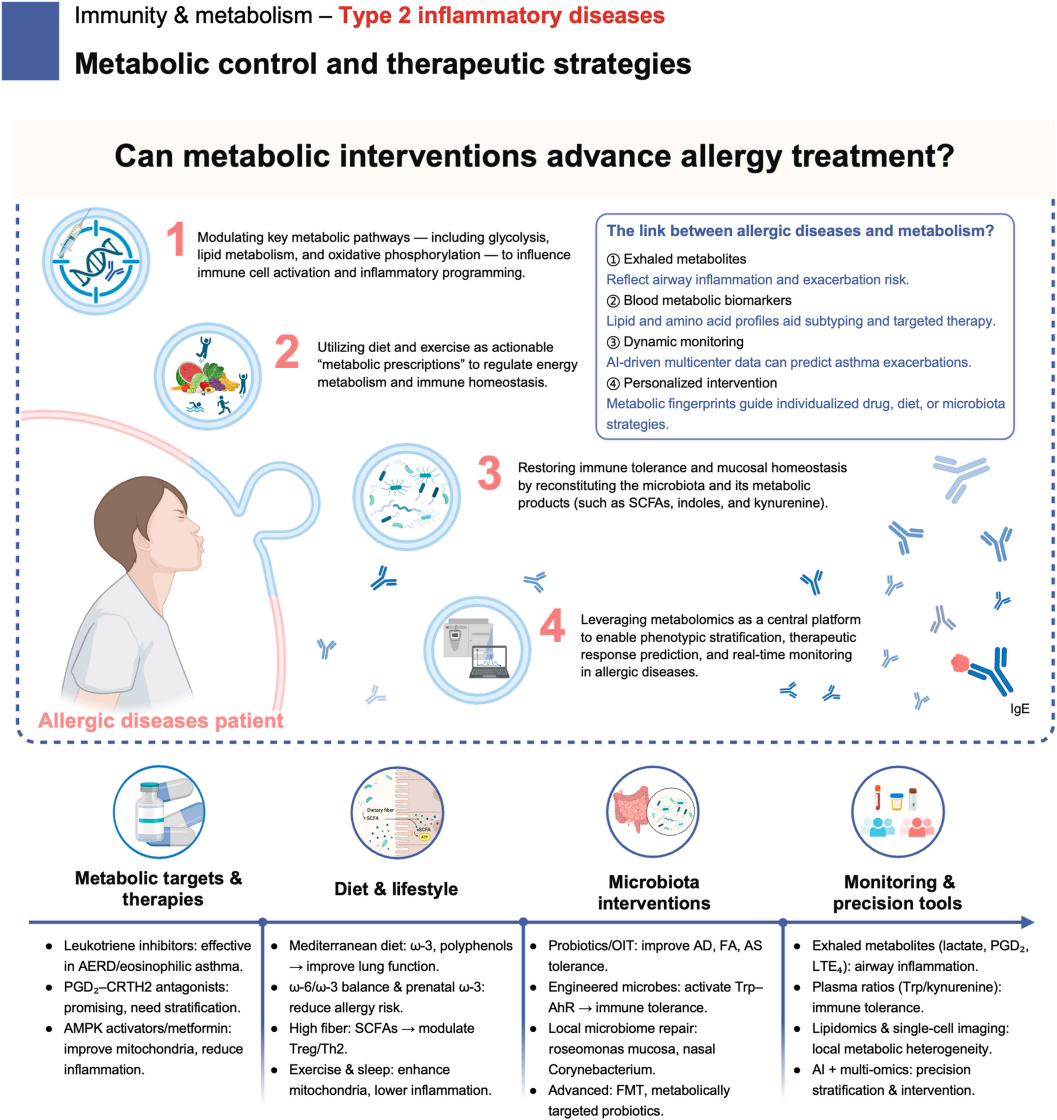
Metabolic control and therapeutic strategies in type 2 inflammatory diseases. ① Metabolic pathway modulation: Targeting glycolysis, lipid metabolism, and oxidative phosphorylation (OXPHOS) to reshape immune activation and tissue inflammation. ② Diet and exercise: “Metabolic prescriptions” such as omega-3 (ω-3) polyunsaturated fatty acids (PUFAs) and polyphenols can improve lung function; exercise and sleep enhance mitochondrial fitness and reduce inflammation. ③ Microbiome restoration: Rebalancing gut flora and metabolites—SCFAs, indoles, and kynurenines—promotes tolerance and mucosal homeostasis; probiotics, engineered microbes, and fecal microbiota transplantation (FMT) show promise in atopic dermatitis (AD), food allergy (FA), and asthma. ④ Metabolomics and precision monitoring: Metabolomics plus artificial intelligence (AI) enables patient stratification and response prediction. Exhaled metabolites—lactate, prostaglandin D₂ (PGD₂), and leukotriene E₄ (LTE₄)—reflect airway inflammation; plasma lipids, amino acids, and tryptophan (Trp)–kynurenine derivatives inform targeted therapy. Integrated interventions: Pharmacologic options include leukotriene inhibitors, PGD₂–CRTH2 (chemoattractant receptor-homologous molecule expressed on Th2 cells) antagonists, and AMP-activated protein kinase (AMPK) activators (e.g., metformin); lifestyle (balanced ω-3/ω-6 intake, high-fiber diet, and regular exercise), microbiota-targeted, and multi-omics/AI-guided precision strategies are emerging.

Treatment of type 2 inflammatory diseases is shifting from terminal anti-inflammation toward coordinated upstream interventions across metabolism, immunity, and the microbiota. Local hypoxia can persistently activate HIF-1α, elevate glycolysis and lactate production, and—via receptors/transporters and histone lactylation—translate metabolic states into inflammatory programs, providing a common, actionable axis for refractory and remodeling-associated phenotypes. Among accessible drugs, the leukotriene pathway remains the most mature paradigm. Clinical use should balance the neuropsychiatric warnings for montelukast and the evidence for urine LTE₄-based stratification and follow-up in AERD [177]. In terms of energy metabolism modulation, AMPK activation—exemplified by metformin—may reduce exacerbations and inflammatory burden, optimize mitochondrial function and the oxidative stress axis, and offer a translational avenue for asthma with metabolic dysregulation. Nutrition and lifestyle can act as “metabolic prescriptions”: High-fiber or SCFA-rich diets down-regulate type II inflammatory responses and MC activation through GPCR/HDAC pathways; prenatal n-3 LC-PUFA interventions associate with lower offspring risk of wheeze/asthma; and regular aerobic exercise with circadian management improves symptoms and nocturnal worsening in randomized or tightly controlled studies [178]. For precision stratification and monitoring, exhaled breath metabolomics combined with machine learning has shown phenotype classification and exacerbation prediction potential in multicenter studies, providing companion diagnostic and response assessment tools for integrated metabolism–immunity–microbiota interventions [179] (Fig. 5).

### Metabolic target therapy strategy

In clinical research and treatment of type 2 inflammatory diseases, exploration of metabolism-related targets is moving from basic studies toward clinical translation, forming a crucial bridge between immune–inflammation control and energy metabolism intervention. In recent years, the metabolic states of immune cells have profoundly shaped their pathogenic functions: Activation of Th2 and ILC2s depends on glycolysis and mTORC1 signaling, while the balance among lipid metabolism, OXPHOS, and FAO in Mφs and Eos determines the persistence and magnitude of inflammation [3,180]. Clinically, the leukotriene pathway is the most mature example of a metabolic target in use: Antagonists of cysteinyl leukotriene receptor 1 (CysLT₁), such as montelukast and zafirlukast, and the 5-lipoxygenase (5-LOX) inhibitor zileuton are applied in asthma, AR, and AERD. Multiple RCTs have shown reductions in exacerbations, improvements in lung function, and nocturnal symptoms, with greater benefits in AERD and eosinophilic asthma. Attention is required to montelukast’s neuropsychiatric adverse warnings and liver-function monitoring with zileuton; in “high lipid-mediator” phenotypes, such as elevated urine LTE₄, these remain safe and effective options [181].

Beyond leukotrienes, the PGD₂–CRTH2 (DP2) pathway is pivotal for chemotaxis of Th2, ILC2s, and Eos. Second-generation CRTH2 antagonists (fevipiprant, timapiprant, GB001) showed phase-2 signals for reducing airway inflammation and improving remodeling; some studies suggested fewer exacerbations or improved quality of life (QoL), yet phase-3 primary endpoints were not met, underscoring the challenges of asthma heterogeneity and therapeutic windows. Future work should re-evaluate biomarker-enriched subgroups—such as high PGD₂, ILC2s-dominant, or eosinophilic asthma—and explore complementarity with ICS or anti-IL-5/anti-IL-4Rα [182].

Modulation of energy pathways also shows promise in preclinical and selected clinical settings: The AMPK activator metformin is associated with fewer exacerbations and reduced oral-steroid use; studies suggest mechanisms that include AMPK–sirtuin 1 (SIRT1) inhibition of NF-κB, partial suppression of mTORC1, reduction of ROS, and restoration of mitochondrial homeostasis, yielding anti-inflammatory and anti-fibrotic effects [183,184]. In severe and refractory phenotypes, mTORC1 inhibitors of the rapamycin class and HIF-1α inhibitors may “break” the vicious cycle of hypoxia–inflammation–remodeling, although risks of systemic immunosuppression limit broad application; local delivery and cell-specific metabolic reprogramming are feasible directions [185].

Emerging avenues include regulation of lipid metabolism (e.g., FASN and ACC inhibitors), coupling of NLRP3 inflammasomes with mitochondrial metabolism, and the role of the SCFA–GPR43 axis in mucosal homeostasis. Translation is advancing toward AD, EoE, and AR, for example, by restoring CER synthesis/barrier function or inhibiting MC degranulation [186]. In summary, metabolic-targeted therapy is promoting a shift from “terminal inflammation control” to “upstream immunometabolic intervention”. The leukotriene pathway provides a successful precedent, while clinical translation of CRTH2, AMPK, mTORC1, and HIF-1α pathways will expand options. Key enablers include precise metabolic subtyping, localized targeted delivery, and robust safety monitoring (Table 5).

**Table 5.** Therapeutic interventions in type 2 inflammation: From conventional mediators to emerging metabolic checkpoints

Target/pathway	Representative drug/strategy	Clinical and mechanistic evidence	Advantages/risks	Future direction
Leukotriene pathway (CysLT₁, 5-LOX)	Montelukast, zafirlukast, zileuton	Multiple RCTs show reduced exacerbations and nocturnal symptoms; stronger signals in AERD and eosinophilic asthma	Mature class, generally safe; neuro-psychiatric AEs for montelukast; LFT monitoring for zileuton	Biomarker-guided use (urinary LTE_4_) and combinations with biologics
PGD₂–CRTH2 (DP2) axis	Fevipiprant, timapiprant, GB001	Phase 2 signals on airway inflammation/QoL; phase 3 primary endpoints not met	Biology clear but population heterogeneity high	Enrich for PGD_2_-high/ILC2-dominant eosinophilic endotypes; combine with ICS or anti-IL-5/IL-4Rα
AMPK pathway	Metformin	Cohorts and preclinical data link to fewer exacerbations and lower inflammatory load; AMPK–SIRT1–NF-κB, partial mTORC1 inhibition, ROS lowering and mitochondrial benefits	Well-tolerated with metabolic benefits	Translational studies in severe asthma with metabolic comorbidity
mTORC1/HIF-1α axis	Rapalogs; HIF-1α inhibitors	Mechanistic studies suggest interruption of the “hypoxia–inflammation–remodeling” loop	Systemic immunosuppression risk	Local delivery and cell-targeted metabolic reprogramming
Lipid metabolism/FAO	FASN, ACC inhibitors	Preclinical evidence for lowering chronic inflammation and remodeling	Preclinical stage	Use multi-omics to stratify metabolic endotypes and target precisely

### Nutritional and metabolic intervention pathways

Nutrition and lifestyle are being re-defined as “clinically actionable metabolic modulators”. Large cohorts (ISAAC, GINI, MAS) indicate that “Western diets” (high fat, high sugar, low fiber) are associated with increased risks of pediatric asthma, AD, and FA; conversely, MD rich in mono/polyunsaturated fatty acids, polyphenols, and antioxidants reduces IL-6 and C-reactive protein (CRP) and improves airway function. Multiple RCTs show that medium- to long-term adherence to MD significantly improves lung function (e.g., FEV₁), enhances asthma control, and reduces the need for inhaled ICS [187].

Optimizing fatty acid composition is pivotal: Supplementation with ω-3 LC-PUFAs such as eicosapentaenoic acid (EPA) and docosahexaenoic acid (DHA) improves AR symptoms in several studies and, during pregnancy, associates with reduced eczema/wheeze in offspring; mechanisms include competitive inhibition of 5-LOX and generation of pro-resolving mediators (resolvins, protectins). In contrast, excessive ω-6 intake elevates prostaglandin and leukotriene production and strengthens the Th2 axis; clinically, maintaining an ω-6/ω-3 ratio < 4:1 is advised [188]. Dietary fiber and the gut ecosystem are central to immune regulation of allergic responses: high-fiber diets increase SCFAs, which via GPR43/41 and HDAC inhibition promote Tregs and suppress Th2, reducing exacerbation frequency and airway inflammation; multicenter/clinical trials show that adding soluble fiber (e.g., inulin/chicory fiber and resistant starch) improves asthma control scores [189].

Regarding lifestyle, regular aerobic exercise activates skeletal-muscle AMPK and peroxisome proliferator-activated receptor γ coactivator 1α (PGC-1α), enhances mitochondrial function and insulin sensitivity, and lowers systemic inflammatory burden; systematic evidence supports ~150 min per week of moderate-intensity activity to improve asthma control, selected lung-function indices, and quality of life. Sleep restriction and circadian disruption worsen nocturnal symptoms by affecting ICS chronobiology, airway inflammation, and autonomic regulation; rigorous rhythm management should be integrated [190]. Looking ahead, a “nutritional metabolic prescription” framework could use metabolomics/microbiome profiling to assess individual “metabolic fingerprints” and tailor diet and lifestyle plans (Table 6).

**Table 6.** Lifestyle-driven immunometabolic modulation: Integrating dietary and behavioral strategies into type 2 inflammation management

Intervention	Primary metabolic/signaling mechanism	Representative evidence	Clinical effect	Limitations/outlook
MD	↓Oxidative stress; lipid mediator modulation	RCTs/cohorts (ISAAC, GINI, MAS) support benefits	Improved FEV₁/ACT and lower ICS use	Highly actionable; adherence and standardization needed
ω-3 LC-PUFA supplementation	5-LOX competition; SPM generation (resolvins/protectins)	Pregnancy supplementation links to lower wheeze/AD risk in offspring; symptom improvement in AR/AD/asthma	Balance ω-6/ω-3 ratio (<4:1) is key	Dose, timing, and responder stratification
High-fiber diet/prebiotics	SCFA–GPR43/HDAC inhibition → ↑Tregs, ↓Th2	Multicenter trials: inulin/resistant starch improve FeNO/ACT	Reduced exacerbations and airway inflammation	Inter-individual variability; metabolomics-guided dosing
Regular aerobic exercise	AMPK–PGC-1α activation; better mitochondrial function and insulin sensitivity	Systematic reviews/RCTs support ~150 min/week moderate activity	Improved asthma control and QoL	Sustained adherence; integrate circadian hygiene
Circadian/sleep management	Aligns ICS rhythms; neuro-immune axes	Night-time worsening linked to circadian disruption	Better nocturnal symptoms and steroid kinetics	Build comprehensive “circadian prescriptions”

### Microbiota–metabolism–allergy regulatory axis

The “microbiota–metabolism–allergy axis” has become a research frontier. Reduced microbiome diversity in infancy is closely associated with increased risks of asthma, AD, and FA; early-life antibiotics, cesarean delivery, and lack of breastfeeding can disrupt homeostasis and delay development of immune tolerance [191]. Probiotics and prebiotics currently have the strongest evidence: Multiple RCTs show that *Lactobacillus rhamnosus* GG (LGG), *Lactobacillus casei*, and *Bifidobacterium breve* have benefits in preventing/treating pediatric FA and alleviating AD; OIT combined with probiotics increases the rate of sustained unresponsiveness; in adult and pediatric AD, LGG/multi-strain probiotics plus standard topical therapy reduce scoring atopic dermatitis (SCORAD) and relapse [192]. Prebiotics such as fructo-oligosaccharides and inulin promote growth of butyrate-producing taxa and enhance SCFA generation, indirectly improving the Tregs/Th2 balance.

More innovative strategies include FMT and “functional microbe” therapies (next-generation probiotics). FMT may promote tolerance in refractory FA, while engineered probiotics aim to restore the Trp–AhR axis and promote tolerance and have entered early clinical testing [193]. Local microbiome repair is also advancing: Topical roseomonas mucosa improves AD inflammation and barrier function in early trials; nasal-microbiome studies suggest associations of Corynebacterium with mucosal phenotypes, and colonization interventions are under exploration [194]. As assays for SCFAs, indoles, and kynurenines mature, precision interventions along the microbiota–metabolism axis may advance from “adding bugs” to “targeted metabolic control” (Table 7).

**Table 7.** Microbiome-based immunometabolic interventions: Restoring ecological diversity and metabolic homeostasis

Modality	Mode of action	Representative studies and conclusions	Advantages	Limitations/future direction
Probiotics (LGG, *B. breve*, *L. casei*)	Restore gut barrier, ↑SCFAs, ↑Tregs	Multiple RCTs show benefits in pediatric AD/FA	Safe and combinable with standard care	Heterogeneous efficacy; need microbiome-matched selection
OIT + probiotics (PPOIT)	Enhance tolerance establishment	Higher rates of “sustained unresponsiveness”	Promising for combination immunotherapy	Long-term safety monitoring required
Prebiotics (inulin/FOS)	Foster butyrate-producing taxa, ↑SCFAs	Improve Treg/Th2 balance	Oral, long-term safety	Dose–response and variability issues
FMT	Rebuild diversity, restore metabolic pathways	Exploratory data: improved tolerance in refractory FA	Holistic micro-ecosystem reset	Standardization/safety still evolving
Engineered probiotics/commensal repair	AhR activation; restore CER synthesis/barrier	Topical roseomonas mucosa improves AD; nasal Corynebacterium exploration ongoing	Targeted metabolic control	Individualized design and longitudinal follow-up

### Metabolic biomarkers and precision diagnosis

Integration of metabolomics, transcriptomics, and microbiomics is pushing metabolic feature-based precision diagnostics and prediction tools into clinical use. Metabolic phenotypes often precede overt inflammation and can sensitively indicate disease activity, treatment response, and relapse risk. In asthma, EBC contains molecules reflecting airway metabolic states—such as lactate, PGD₂, LTE₄, and oxidized lipid metabolites—whose levels correlate with lung function and disease control; some studies link them to exacerbations and inflammatory burden [195]. In addition, plasma Trp:kynurenine ratios are being explored in allergy-related studies and immunotherapy monitoring; lower or imbalanced ratios indicate immune activation with insufficient tolerance [196].

In AR and AD, lipidomic and amino acid signatures reveal pathway-specific abnormalities that inform stratification and targeted intervention. At single-cell and spatial levels, metabolic/transcriptional imaging further resolves local airway metabolic heterogeneity and guides local drug delivery [197]. Artificial intelligence and machine learning can integrate metabolic data; multicenter validation and systematic studies show potential for predicting asthma exacerbations. VOC-based technologies such as e-nose are entering clinical validation and may merge with point-of-care testing (POCT) [198]. Ultimately, integrating metabolomics, microbiomics, genomics, and clinical phenotypes may establish a “metabolism-driven precision-medicine loop”, using metabolic fingerprints for risk prediction and response monitoring to guide personalized nutrition, drugs, and microbial interventions—enabling dynamic, closed-loop management from prevention to diagnosis and therapy. Examples include ICS adjustment based on exhaled breath metabolic fingerprints, local lipid-replenishment or anti-inflammatory regimens guided by skin metabolic imaging, and adaptive protocol tuning during OIT according to metabolic profiles (Table 8).

**Table 8.** The next frontier in precision diagnostics: Leveraging immunometabolic biomarkers and multi-omics integration

Biomarker/modality	Pathway or meaning	Clinical use/findings	Technical advantages	Direction of travel
EBC lactate, PGD_2_, LTE_4_	Local glycolysis and lipid-mediator activity	Correlate with lung function, control and inflammatory burden; signals for exacerbation risk	Noninvasive, longitudinal monitoring	AI pairing to predict exacerbations and treatment response
Plasma Trp/kynurenine ratio	IDO activity and tolerance state	Imbalance signals inflammation; explored in immunotherapy monitoring	Simple blood test	Define reference intervals and dynamic monitoring schemes
Lipidomic/amino acid signatures	AR/AD endotypes	Sub-phenotype discrimination and therapy guidance	Fine-grained stratification	Integrate with single-cell/spatial omics for local delivery cues
VOCs/e-nose	Composite metabolic fingerprints	Multicenter validation suggests potential for predicting asthma exacerbations	POCT-ready	Bedside monitoring and ICS titration guidance
Integrated multi-omics diagnostics	Metabolome + microbiome + genome	Risk prediction, response assessment and intervention guidance	Precision “closed loop”	Build metabolic-driven precision-care systems

## Conclusion and Perspectives

Research on type 2 inflammatory diseases is shifting from a singular paradigm of “immune imbalance” toward a new framework of “immune–metabolic crosstalk”. Energy metabolism is not only the “fuel” for immune responses but also a key signaling hub that determines immune cell fate and function. The dynamic balance among glucose, fatty acid, and amino acid metabolism jointly shapes activation, differentiation, and tolerance thresholds of cells, with elevated glycolysis typically accompanying augmented proinflammatory output from effector cells such as Th2 and ILC2s. FAO and OXPHOS help maintain the homeostasis of Tregs and M2-like Mφs. Trp, Gln, and Arg metabolism, via pathways including IDO–AhR–mTOR, finely sets the boundary between immune tolerance and inflammatory amplification. This “metabolism–immunity” crosstalk network provides new theoretical and actionable coordinates for understanding the initiation and chronicity of allergic responses.

At the cellular level, metabolic reprogramming spans the entire course of allergic reactions. Effector populations—including Th2, ILC2s, Eos, MCs, and PCs—commonly display high glycolysis and heightened oxidative stress, whereas the metabolic states of DCs, Mφs, and ECs in antigen presentation, barrier maintenance, and tissue remodeling determine the extent of inflammatory propagation and terminal structural change. The distinct “metabolic fingerprints” of different cell populations together constitute the metabolic ecosystem of allergic inflammation, whose homeostasis or imbalance directly affects disease severity and therapeutic responsiveness. Tissue-specific metabolic microenvironments further amplify these differences: In the airways, the hypoxia–HIF-1α–glycolysis–lactate axis synergizes with epithelial “alarmin” networks to sustain asthma chronicity; in the skin, imbalance in CER and FFA profiles weakens the barrier and amplifies type II immunity; in the gut, microbiota-derived SCFAs and indole metabolites gate the transition between tolerance and sensitization.

Multi-omics technologies are advancing the delineation of a “metabolic reprogramming atlas” of type 2 inflammatory diseases. Metabolomic, transcriptomic, and spatial single-cell data reveal that different conditions—such as asthma, AR, AD, FA, and CRSwNP—share common pathways like the mTORC1–glycolysis axis while exhibiting tissue-specific signatures in lipid or amino acid metabolism. This underpins “metabotype–phenotype” stratification and enables incorporation of metabolic information into response prediction and treatment decisions. On the translational front, leukotriene pathway inhibitors montelukast and zileuton first demonstrated the feasibility of “metabolism–lipid mediator”-based intervention. CRTH2 antagonists, AMPK activators, and HIF-1α inhibitors are under clinical evaluation, while mTOR inhibitors, metabolic enzyme modulators, and cell-specific nano-delivery systems are opening routes to highly specific, low-toxicity metabolic interventions. In parallel, “metabolic prescriptions” such as MD, ω-3 fatty acid supplementation, high-fiber diets, and regular exercise, by improving metabolic homeostasis and the microbiome, have been incorporated into comprehensive management for selected conditions; probiotics, prebiotics, FMT, and engineered “functional microbes” show promise for rebuilding the “microbiota–metabolism–immunity” axis.

Looking ahead, “metabolically precise allergology” will become a key direction. Single-cell and spatial metabolomics can map lesion microenvironments to resolve cell–cell metabolic interactions; integrative models combining artificial intelligence with multi-omics are expected to reconstruct dynamic networks of “metabolism–genetics–microbiota–environment” for individualized risk prediction and response assessment; metabolic biomarkers, together with portable sensors and rapid mass spectrometry, will drive the routine clinical adoption of bedside metabolic monitoring. More importantly, deepening metabolic research will promote a therapeutic transition from pure immunosuppression to dual-axis resetting of metabolism and immunity—from passive “inflammation control” to proactive “restoration of homeostasis”.

Overall, type 2 inflammatory diseases should be viewed as systemic disorders arising from coordinated disequilibrium among energy metabolism, cellular signaling, and environmental exposures. When each patient’s “metabolic–immune fingerprint” can be precisely delineated and jointly calibrated through pharmacologic, nutritional, exercise, and microbiota interventions, the diagnosis and treatment of allergology will truly enter the era of “metabolically precise medicine”. This will not only reshape clinical practice but also serve as an exemplar of how immunometabolism can drive the implementation of precision medicine.

## Data Availability

No datasets were generated or analyzed during the current study.
